# NtMYB1 and NtbZIP53 antagonistically regulate NtGA3ox2 and NtGA2ox8 mediating diverse GA biosyntheses and tiny-plant in *Narcissus tazetta*

**DOI:** 10.1093/hr/uhag234

**Published:** 2026-06-08

**Authors:** Zhuoyu Wu, Xi Wu, Zheng Gu, Jingwen Yang, Yajue Chen, Yun Xu, Ruizhe Feng, Yu Zhang, Yujun Ren, Ying Miao

**Affiliations:** Fujian Key Laboratory of Plant Functional Biology, Fujian Agriculture and Forestry University, Fuzhou 350002, China; Fujian Key Laboratory of Plant Functional Biology, Fujian Agriculture and Forestry University, Fuzhou 350002, China; Fujian Key Laboratory of Plant Functional Biology, Fujian Agriculture and Forestry University, Fuzhou 350002, China; Institute of Forestry and Pomology, Wuhan Academy of Agricultural Sciences, Wuhan 430070, China; Fujian Key Laboratory of Plant Functional Biology, Fujian Agriculture and Forestry University, Fuzhou 350002, China; Fujian Key Laboratory of Plant Functional Biology, Fujian Agriculture and Forestry University, Fuzhou 350002, China; Fujian Key Laboratory of Plant Functional Biology, Fujian Agriculture and Forestry University, Fuzhou 350002, China; Fujian Key Laboratory of Plant Functional Biology, Fujian Agriculture and Forestry University, Fuzhou 350002, China; Fujian Key Laboratory of Plant Functional Biology, Fujian Agriculture and Forestry University, Fuzhou 350002, China; Fujian Key Laboratory of Plant Functional Biology, Fujian Agriculture and Forestry University, Fuzhou 350002, China

## Abstract

Miniaturization and multiple branching are characteristic features of horticultural plants. Gibberellins participate in regulating the size and branching of plant architecture. Gibberellin 3β hydroxylase (GA3ox) and gibberellin 2β hydroxylase (GA2ox) are a pair of key enzymes for the homeostasis of active GA. However, the transcriptional regulation of GA3ox and GA2ox and the synthesis of active GAs remain unknown. Here the roles of narcissus NtMYB1 and NtbZIP53 in regulating the biosynthesis of diverse active GAs were addressed. Overexpression of *NtMYB1* in *Arabidopsis* and *Narcissus* shows a tiny-type phenotype. The *NtMYB1* co-expression network reveals that *NtMYB1* or *NtbZIP53* is closely related to *NtKS1, NtGA20ox*, *NtGA2ox*, and *NtGA3ox* in the GA biosynthetic pathway of narcissus. NtMYB1 can interact with NtbZIP53. Overexpression of *NtMYB1* and *NtMYB1*/*NtbZIP53* in transgenic narcissus upregulated the expression of upstream genes *NtKS1* and *NtGA20ox* in the GA biosynthesis pathway; however, NtMYB1 and/or NtbZIP53 directly targeted the promoters of *NtGA3ox2* and *NtGA2ox8* and antagonistically controlled *NtGA3ox2* and *NtGA3ox8* transcription, respectively, and both decreased GA_1_ accumulation. NtMYB1 recruiting NtbZIP53 enhanced the repression of *NtGA3ox2* and the activation of *NtGA2ox8*, reducing the GA_1_/GA_3_ ratio and plant size in narcissus. This provides a conceptual basis for controlling the architecture of Chinese narcissus.

## Introduction

Horticultural plant architecture holds significant ecological and economic value. Miniaturization and multiple branching are characteristic features. *Narcissus tazetta* var. *chinensis* cv. ‘Jinzhanyintai’ belongs to the Amaryllidaceae family and is a perennial bulbous plant widely cultivated in East Asia and China. It is one of the 10 most renowned traditional flowers in China, renowned for its beautiful and highly ornamental flowers with a rich fragrance. However, its simplistic plant architecture limits its commercial appeal.

Gibberellins (GAs) are a class of tetracyclic diterpenoid phytohormones that regulate multiple aspects of plant development. Although large numbers of different GA species have been identified, only a few, such as GA_1_, GA_3_, GA_4_, and GA_7_, possess biological activity [1, 2]. The other forms of GA present in plants are either precursors of the bioactive forms or deactivated metabolites. GA_1_ and GA_4_ are the two major active GAs in most plant species, although GA_3_ has also been detected in plants. The bioactivity of GA_4_ is significantly higher than that of GA_1_ in both *Arabidopsis* and rice, which is presumably attributable to their differences in binding affinity to the GA receptor [3–6]. Different plant species have diverse ratios of GA_1_, GA_4_, and GA_3_ in various tissues. The most prominent functions of GAs include promoting stem elongation, leaf expansion, flowering, fruit development, and seed germination, and influencing plant architecture [7, 8]. Plants deficient in GA biosynthesis or the GA signaling pathway display characteristic phenotypes, such as dwarfism, small dark-green leaves, prolonged germination, root growth retardation, suppression of flowering, reduced seed production, and male sterility [9, 10].

The biosynthesis of GAs originates from a branch of the diterpenoid pathway. It can be divided into three major stages based on the localization and nature of the enzymes involved [11, 12]. The formation of GAs begins with the cyclization of geranyldiphosphate in the plastid. Subsequently, GA_12_-aldehyde is formed by the action of cytochrome P450-dependent mono-oxygenases on the exterior of the endoplasmic reticulum. GA_12_-aldehyde is the first GA produced and serves as the precursor of all other GA species. They are formed in the cytosol in a complex metabolic network that varies among species and developmental stages, and in a tissue-specific manner. The later steps of the pathway are catalysed by soluble 2-oxoglutarate-dependent dioxygenases. This group comprises enzymes either involved in the formation of the bioactive GA_1_ and/or GA_4_ by GA_20_-oxidases and GA_3_-oxidases or involved in their deactivation by GA_2_-oxidases. Both GA_3_-oxidase and GA_2_-oxidase have been regarded as important regulators of the biosynthetic pathway in biochemical and molecular studies [11–13]. GA3ox can oxidize non-bioactive GA_12_ and GA_53_ to the bioactive GA_3_ and GA_1_. In contrast, GA2ox can oxidize the bioactive GA_4_ to non-bioactive GA_8_ in rice [2, 7].

It has been reported that the transcription of GA biosynthesis genes *GA3ox1*/*2* and *GA20ox2* and the GA metabolic gene *GA2ox* is affected by DELLA proteins, which are members of the GRAS family in *Arabidopsis* [14], and accumulate in the nucleus [15, 16]. DELLA proteins activate the expression of *AtGA20ox2* and *AtGA3ox1* genes, but there has been no direct evidence showing that DELLA proteins bind to the *AtGA20ox2* and *AtGA3ox1* promoter. Overexpression of *YAB1* resulted in a semi-dwarf phenotype with decreased *OsGA3ox2* transcript levels, whereas co-suppression of *YAB1* in transgenic plants induced expression of *OsGA3ox2*. YAB1 binds to the GA-responsive element (GARE) located in the *GA3ox2* promoter, indicating that YAB1 regulates *OsGA3ox2* directly [17]. The GARE sequence does not exist in the *OsGA20ox2* promoter. Later, an RSG binding sequence (rbe) in the promoter region of *NtGA20ox1* was identified. RSG, a bZIP transcription factor, binds to this sequence of the *NtGA20ox1* promoter *in vivo* in response to a decrease in GA levels, and the binding is abolished by exogenous GA treatments [18, 19]. Therefore, RSG not only controls the expression of *NtKS* [20], but also the feedback regulation of *NtGA20ox1* [19]. Further studies in transgenic tobacco plants indicated that SbMYB2 and SbMYB7 might regulate secondary biosynthesis through the GA pathway [21]. In previous research, based on comparative transcriptomic and metabolite analysis during narcissus development, the co-expression network of transcription factor and structural genes showed that *NtMYB1*, *NtbZIP*, and *NtGRAS* co-expressed with *DXR*, *NCED*, and *PDS* genes during narcissus development [52, 53]. Overexpressing *NtMYB1* increased the contents of ABA, zeatin,□β-carotene, and zeaxanthin, but reduced the GA1 content and increased the GA3 content, resulting in an early petal senescence phenotype in narcissus and a mini-type plant in *Arabidopsis* overexpressing NtMYB1 [52]. However, how the mechanisms of MYB1, bZIP, and GRAS affect the biosynthesis of the diverse GAs in various plants is unclear, particularly in narcissus plants.

In this study, we identified both *NtMYB1* and *NtbZIP53* genes from *Narcissus tazetta*. We further generated *oeNtMYB1*, *oeNtbZIP53*, and both *oeNtMYB1* and *oeNtbZIP53* in narcissus and transgenic *Arabidopsis* plants. Meanwhile, based on weighted gene co-expression network (WGCNA) analysis, several candidate targeted genes were selected, the promoter sequences of *NtGA3ox2* and *NtGA2ox8* were isolated by using a genome walking assay, and then we biochemically investigated the mechanism of NtMYB1 and NtbZIP53 in regulating the production of diverse GAs in narcissus. This provides a fundamental basis for controlling narcissus ‘mini’ architecture breeding.

## Results

### Overexpressing *NtMYB1* in *Arabidopsis* and narcissus produced a GA-deficient-like dwarfism plant phenotype

In a previous report, the overexpression of *NtMYB1* in *Arabidopsis* and narcissus manifested an accelerating leaf and petal senescence phenotype [52]. Additionally, a tiny plant type including a short root, hypocotyl, petiole, and rosette area and a late-flowering plant phenotype as well as higher chlorophyll *b* content were observed in *Arabidopsis* plants overexpressing *NtMYB1* (Supplementary Material Fig. S1). Further systematic phenotyping in narcissus revealed a tiny-bulb phenotype in *oeNtMYB1-GFP* transgenic narcissus plants in the *T*_1_ generation compared with the wild-type (WT) and oeGFP transgenic narcissus plant (Fig. 1A). Considering that the tiny phenotype of *oeNtMYB1* transgenic lines might stem from reduced GA biosynthesis, an exogenous GA treatment would rescue the tiny phenotype induced by *NtMYB1* overexpression. To test this hypothesis, we administered exogenous GA_3_ to transgenic *Arabidopsis* and narcissus bulbs, which promptly increased the endogenous GA_1_ and GA_3_ contents of treated leaves compared with the mock (Fig. 1B; Supplementary Material Fig. S2). The expression level of the GA receptor gene *NtGID1* was promoted in the WT and oeGFP alone narcissus bulb after GA_3_ treatment; however, overexpressing NtMYB1 reduced the transcript level of *NtGID1* to the same level, and even exogenous application of GA_3_ did not alter the *NtGID1* expression level (Fig. 1C). The tiny-plant phenotype of the *oeNtMYB1* narcissus bulb and *Arabidopsis* partially recovered after 4 weeks of growth (Fig. 1A; Supplementary Material Fig. S2), indicating that overexpression of *NtMYB1* reduced the GA_1_ level, resulting in a mini-bulb, GA-deficient like dwarfism plant phenotype, which can be partially rescued by exogenous GA_3_ application.

**Figure 1:**
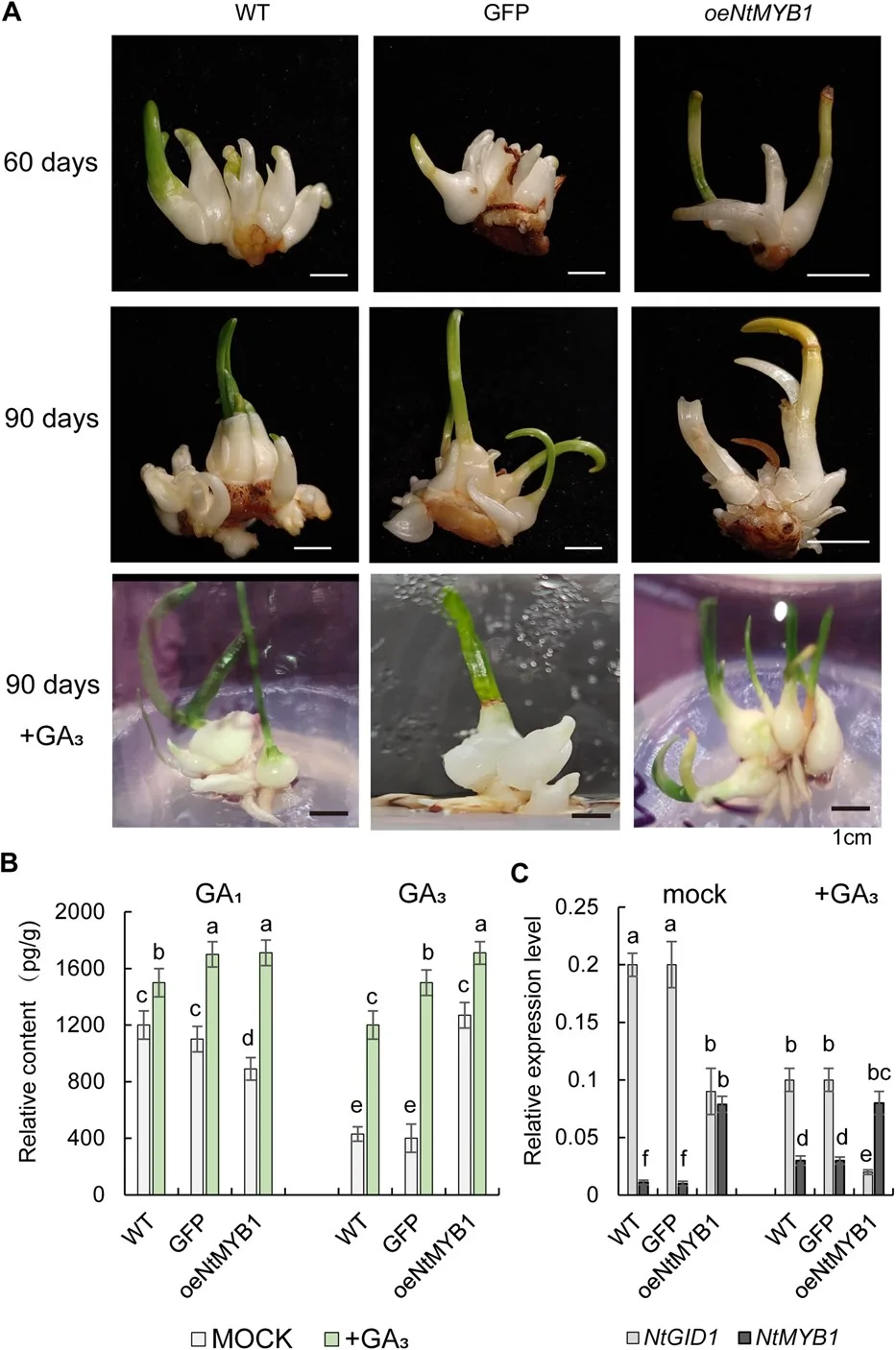
*oeNtMYB1* transgenic narcissus in different development stages showed a mini-bulb phenotype. (A, B). Mini-bulb phenotype at 60 d (A) and 90 d (B); (C) 60-d transgenic bulb and WT bulb after treatment with 100 μM gibberellin (GA_3_) in the medium for 4 weeks. (D) Contents of GA_1_ and GA_3_ compared with WT after treatment for 24 h. (E) GA receptor gene *NtGID1* expression level of the tissue treated with GA_3_ for 24 h compared with mock (normal medium) by RT–qPCR. Values are averages of three biological replicates. Statistical analysis was by Tukey’s test; different letters indicate significance at *P* < 0.05.

### Overexpression of *NtMYB1* alters diverse gibberellin contents in narcissus

The influence of *NtMYB1* overexpression on the phenotype was hypothesized that *NtMYB1* might represses the GA biosynthesis pathway. To test this, we measured the contents of diverse GAs, including GA_12_, GA_53_, GA_20_, GA_5_, GA_1_, GA_3_, and GA_4_, in the control and transgenic lines. The GA_1_ content of the transgenic lines was significantly lower than that of control plants (Fig. 2), with the maximum repression in plant size observed in transgenic narcissus and *Arabidopsis* (Fig. 1; Supplementary Material Fig. S2). As expected, the degree of reduction in GA_1_ content in narcissus followed the degree of overexpression of *NtMYB1* (Fig. 2A). However, the content of GA_3_ in different transgenic lines was significantly increased, even though the level of GA_3_ was much lower than the level of GA_1_ in narcissus leaves, while GA_4_ was undetectable in leaves (Fig. 2B and C). Similarly, the contents of GA_12_ and GA_53_ were also augmented in the transgenic narcissus lines overexpressing *NtMYB1* (Fig. 2B and C). Thus, it seems that the tiny phenotype caused by overexpressing *NtMYB1* is attributable to the decline in the GA_1_/GA_3_ ratio in narcissus leaves (Fig. 2D).

**Figure 2:**
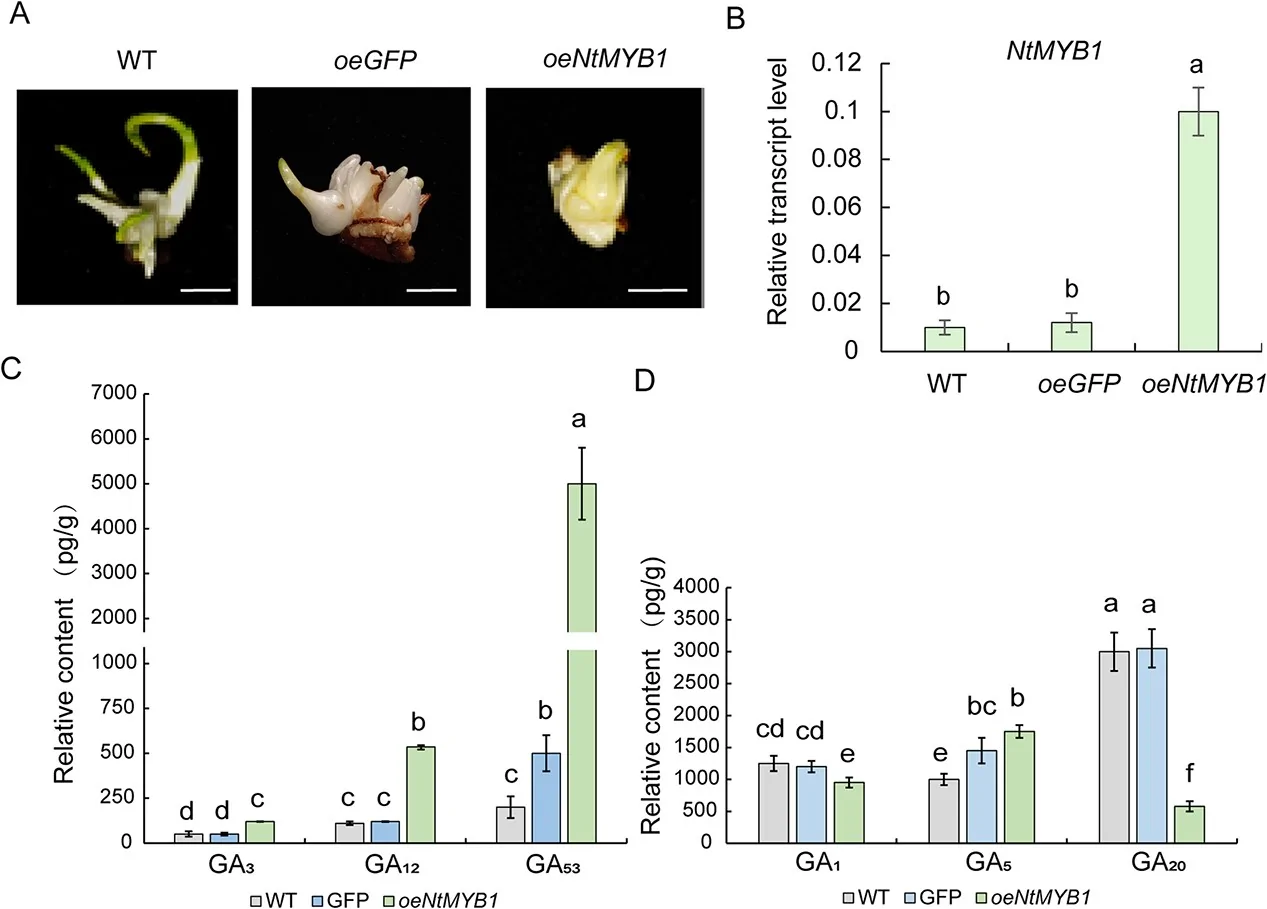
Hormone contents in *oeNtMYB1* transgenic narcissus and WT. (A) 60-d-old narcissus bulbs. Scale bar = 0.5 cm. (B) Transcript level of *NtMYB1* in *oeNtMYB1* narcissus bulb relative to WT and *oeGFP*-alone bulbs. (C) The contents of different GA species: GA_3_, GA_12_, GA_53_; (D) The contents of different GA species: GA_1_, GA_20_, GA_5_. Statistical analysis was by Tukey’s test; different letters indicate significance at *P* < 0.05.

### *NtMYB1* and *NtbZIP53* co-expressed with *NtGA3ox* and *NtGA2ox* in *Narcissus tazetta*

Referencing the GA biosynthesis pathway in *Arabidopsis* and rice, GA biosynthetic pathway-related enzyme genes, including *CPS*, *KS1*, *KO*, *KAO*, *GA3ox*, *GA20ox*, and *GA2ox*, were selected from *Arabidopsis* (Fig. 3A; KEGG pathway). The previous transcriptome dataset (NBCI, PRJNA750844, https://phytozome.jgi.doe.gov) of *N. tazetta* during flower development was employed for alignment analysis [22, 23] and produced a series of GA biosynthetic pathway-related enzyme genes (195 genes) in narcissus (Supplementary Material Dataset S1), and a putative GA biosynthesis pathway in narcissus was established (Fig. 3B). Then, the co-expression network of the transcription factors with GA enzyme genes from the narcissus transcriptomic dataset was analysed by WGCNA, a common method for constructing gene co-expression networks [23, 24]. According to the thresholds (|GS| > =0.2;|MM| ≥ 0.8, *P*-values <0.05), six modules of transcription factors were produced during narcissus development (Supplementary Material Fig. S3). We analysed the co-expression networks of all transcription factors from these six modules and all the selected 195 genes related to GA metabolism by WGCNA. Several transcription factors, including GRAS, C2H2, bZIP, WRKY, MYB1, ARF, TUB, HB, and GARP-G2, are involved in the GA metabolism pathway at different development stages of *N. tazetta* (Fig. 3C). Among them, NtbZIP (c145326) is 52.08% homologous to AtbZIP53, which is designated as NtbZIP53 (Supplementary Material Fig. S4). NtMYB1 and NtbZIP53 were strongly related to NtGA20ox (c110461), NtGA3ox2(c62538), and NtGA2ox8 (c109460) enzymes. The relative intensity is 0.79 (Supplementary Material Fig. S5), where GA20ox and GA3ox are key restrictive enzymes for non-active GA12 or GA53 transferring to active species GA_1_, GA_4_, and GA_3_ in *Arabidopsis* and rice, while GA2ox is a key enzyme for the deactivation of active GAs [7]. Thus, both NtMYB1 and NtbZIP53 might be implicated in the regulation of *NtGA20ox1*, *NtGA3ox1*/*2*, and *NtGA2ox8* expression in narcissus*.*

**Figure 3:**
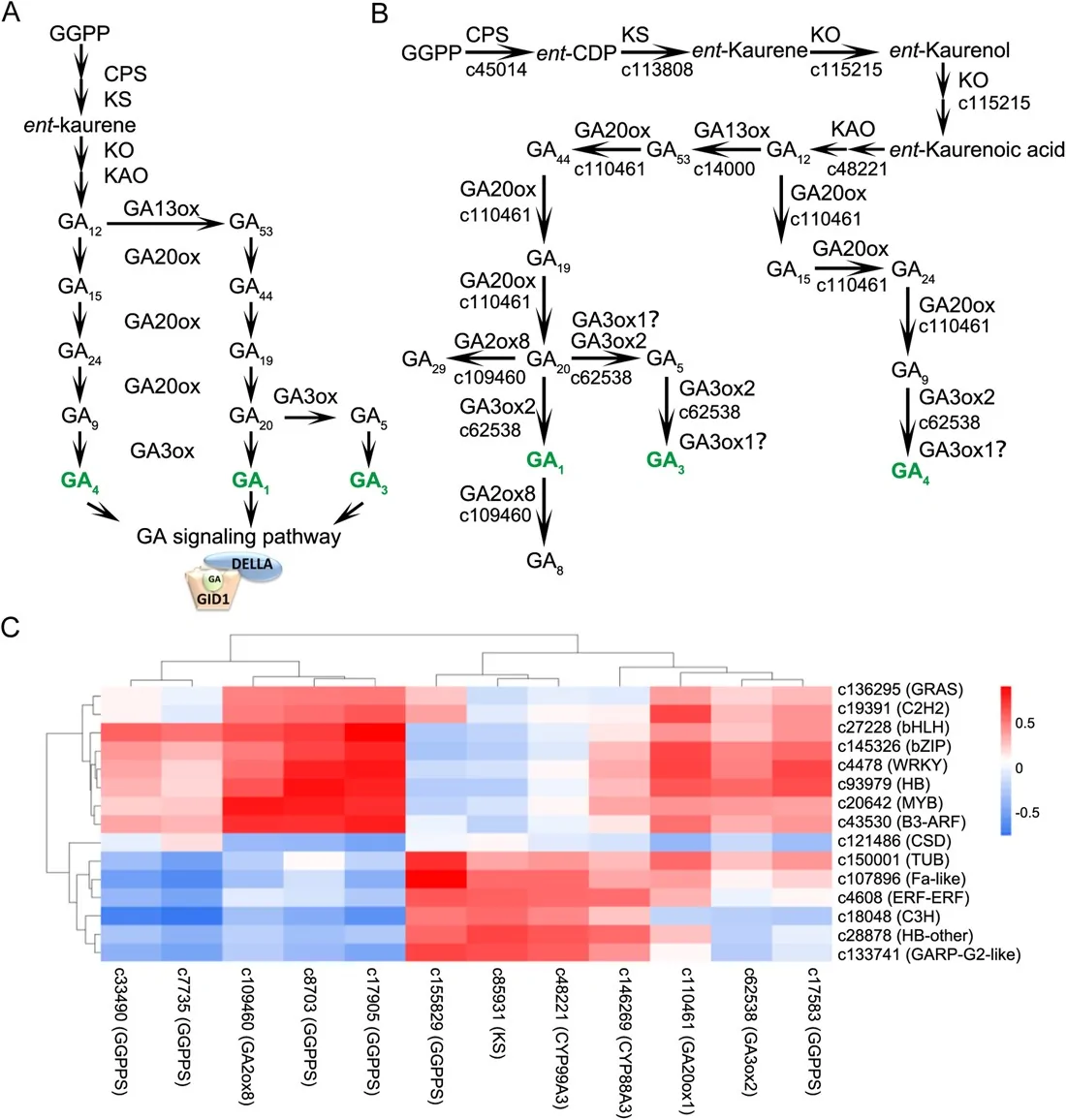
*NtMYB1* and *NtbZIP53* co-expressed with *NtGA20ox*, *NtGA3ox*, and *NtGA2ox* during narcissus flower development. (A) Brief GA biosynthesis pathway in *Arabidopsis* and rice. (B) Putative GA biosynthesis pathway in *Narcissus*. (C) Co-expression network of several transcription factors with GA biosynthetic enzymes in *Narcissus*. The datasets come from the transcriptome dataset (NBCI, PRJNA750844, https://phytozome.jgi.doe.gov) of *Narcissus tazetta* during flower development in our previous work). Red color indicates a positive relation; blue color indicates a negative relation.

### NtMYB1 interacts with NtbZIP53 in the nucleus

To test the relationship among the three transcription factors NtMYB1, NtbZIP53, and NtGRASthat belong to the same family as DELLA proteins, the interaction among three candidate proteins, NtbZIP53, NtGRAS, and NtMYB1, was examined by yeast two-hybrid (Y2H), bimolecular fluorescence complementation (BiFC), and co-immunoprecipitation (Co-IP) assays. The Y2H results indicated that yeast strain AH109 co-transformed with plasmids AD-NtbZIP53 and BD-NtMYB1 grew well in medium SD minus amino acids Leu, Ade, Trp, and His, while colonies co-transformed with an AD-NtMYB1 with BD-NtGRAS and AD vector with BD-NtMYB1 did not grow infour amino acids deficient medium (SD minus amino acids Leu, Ade, Trp, and His), suggesting that NtMYB1 can interact with NtbZIP53 in yeast cells (Fig. 4A); however NtMYB1 or NtbZIP53 did not interact with NtGRAS (Supplementary Material Fig. S6). The localization of NtbZIP53 was further detected in the nucleus of protoplasts of narcissus leaf (Fig. 4B). Next, we confirmed the interaction of NtbZIP53 and NtMYB1 in epidermal cells of tobacco leaf by a BiFC assay. When plasmids NtMYB1-YFPc and NtbZIP53-YFPn were co-injected into epidermal cells of tobacco leaves, a YFP fluorescence signal emerged in the nucleus (Fig. 4C). However, when NtMYB1 or NtbZIP53 was co-transformed with empty negative control, the YFP fluorescence signal did not appear in the cell (Fig. 4C). Furthermore, pulldown of co-transformed tobacco leaf with oeNtMYB1-HA, oeNtbZIP-GFP plasmids using GFP antibody was immunodetected with antibody against HA. This result showed that NtMYB1-HA can be detected in the immunoprecipitate (IP) (Fig. 4D). This demonstrated that NtMYB1 can physically interact with NtbZIP53 (Fig. 4D).

**Figure 4:**
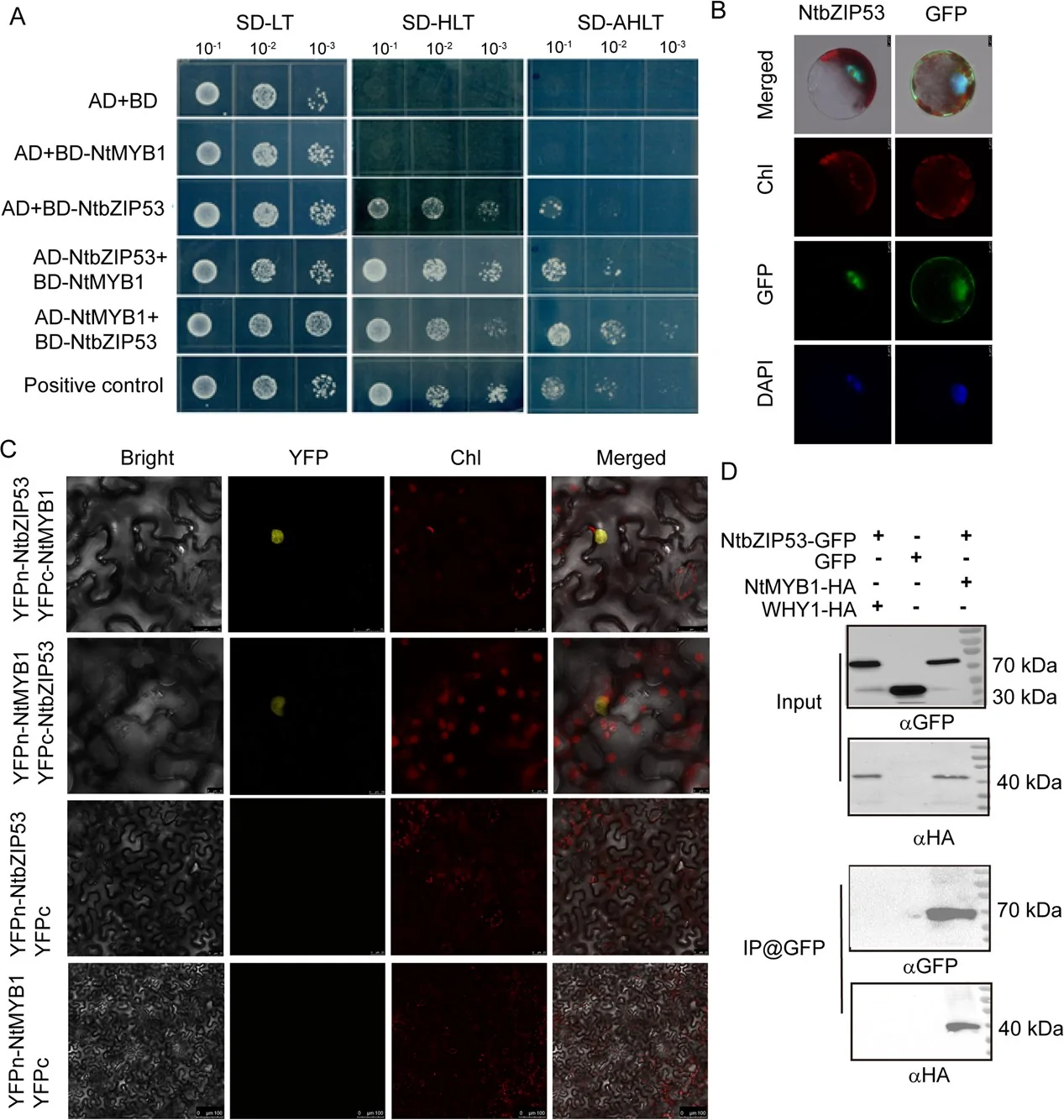
Identification of the interaction of two candidate proteins, NtMYB1 and NtbZIP53. (A) NtMYB1 interacts with NtbZIP53 in yeast cells. Scale bars = 1 cm. (B) NtbZIP53 localized in the nucleus of narcissus protoplast. Scale bars = 5 μm. (C) NtMYB1 interacts with NtbZIP53 in the nucleus of tobacco epidermal cells by BiFC assay. Scale bars = 20 μm. (D) Co-immunoprecipitation detection of NtMYB1-HA with NtbZIP53-GFP by using antibody against GFP; antibody against HA was used to immunodetect IP.

### NtMYB1 and NtbZIP53 antagonistically regulated *NtGA3ox1/2* and *NtGA2ox8* expression

Next, to explore the regulatory roles of both NtMYB1 and NtbZIP53 in the GA biosynthetic pathway, initially we generated and identified overexpression of *NtbZIP53* and overexpression of both *NtMYB1* and *NtbZIP53* in narcissus lines and *Arabidopsis atbzip53* mutant lines (Supplementary Material Figs S7 and S8). Based on the intensity of the relation rate (relative rate >0.79) of the co-expression network, we selected *NtKS1*, *NtGA3ox1*, *NtGA3ox2*, *NtGA20ox*, *NtGA2ox2*, and *NtGID1* genes of the GA biosynthetic pathway in narcissus tissue (Fig. 3C) as candidates to examine their expression levels in transgenic narcissus overexpressing *NtMYB1* and *NtbZIP53*. Quantitative RT–PCR analysis revealed significant alterations in the expression levels of *NtKS1*, *NtGA3ox1*/*2*, *NtGA2ox2*, and *NtGID1* with various fold changes. The expression of *NtKS1* increased by 1.5- to 2-fold in the *oeNtMYB1* and *oeNtbZIP53* narcissus and by 3-fold in *oeNtMYB1*/*NtbZIP53* narcissus compared with WT (Fig. 5A); the expression of *NtGA20ox* increased by 20-fold in *oeNtMYB1* and *oeNtbZIP53* and by 25-fold in *oeNtMYB1*/*NtbZIP53* narcissus relative to WT (Fig. 5B); the expression of *NtGA3ox2*, *NtGA2ox2*, and *NtGID1* was down-regulated by 4-fold in *oeNtMYB1* and *oeNtMYB1*/*NtbZIP53*, but was upregulated by 3-fold in the *oeNtbZIP53*, in comparison with the WT. In contrast, the expression of *NtGA3ox1* increased by 20-fold in *oeNtMYB1* and *oeNtMYB1*/*NtbZIP53*, as well as in *atbzip53* mutant, yet remained unaltered in *oeNtbZIP53*, compared with the WT (Fig. 5C–F). These results suggested that overexpression of both *NtMYB1* and *NtbZIP53* in narcissus has the potential to modify the expression of genes such as *NtKS1-* and *NtGA20ox1* involved upstream in the GA biosynthesis pathway; however, NtMYB1 and NtbZIP53 antagonistically affected the expression of *NtGA3ox1* and *NtGA3ox2*, respectively, as well as *NtGA2ox2*. NtMYB1 predominantly repressed the expression of *NtGA3ox2* but activated that of *NtGA2ox2* and *NtGA30x1*, while *oeNtbZIP53* had no effect on that of *NtGA3ox*1. In addition, the overexpression of *NtbZIP53* in *Arabidopsis* knockout line *atbzip53* could rescue the phenotype of *atbzip53* (large size of plant) so that it was like WT plants (Fig. 5G). This suggests that NtbZIP53 has conserved function with respect to AtbZIP53, being involved in the regulation of plant size.

**Figure 5:**
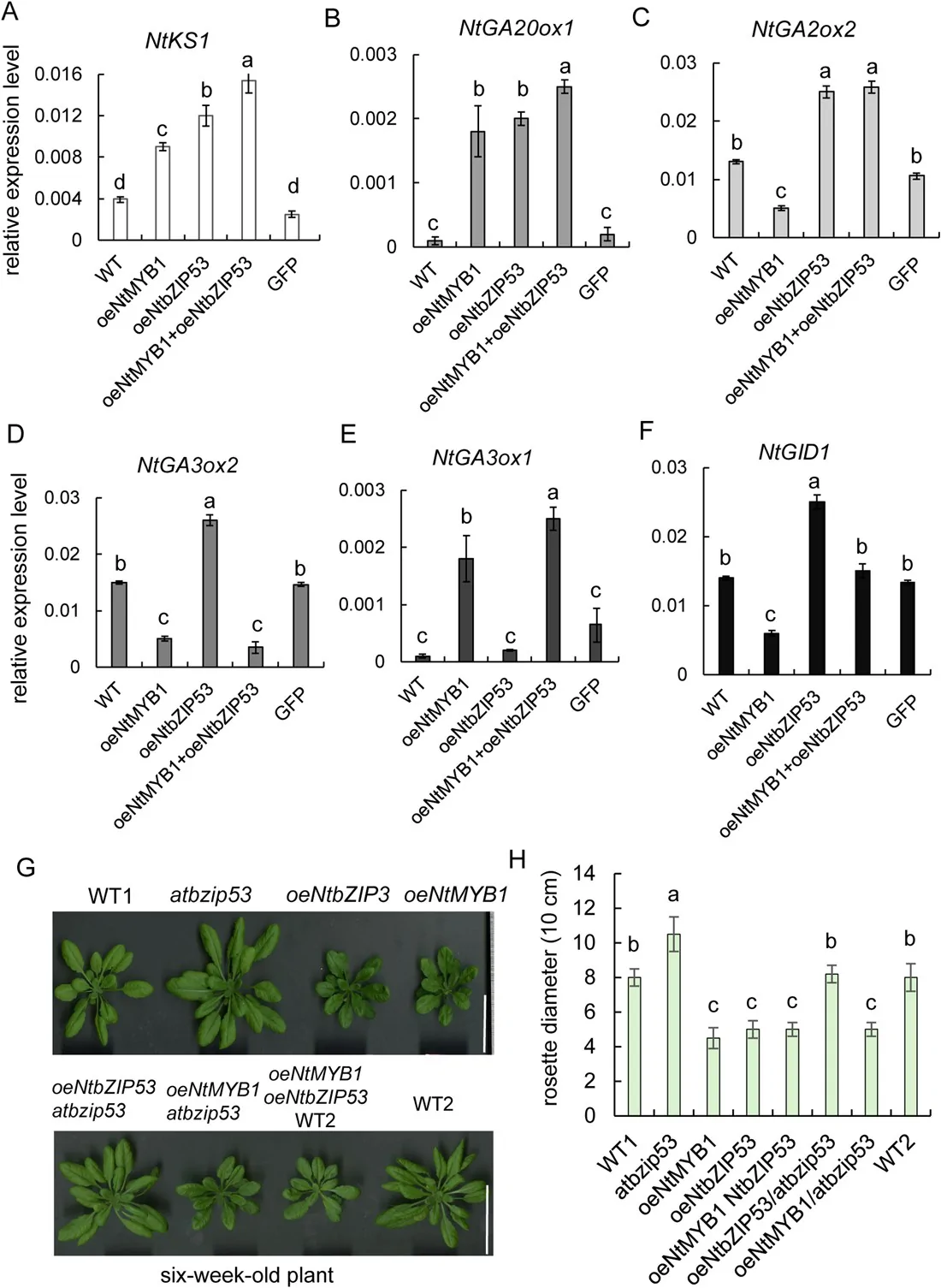
Gene expression profiles of GA biosynthetic enzymes in *oeNtMYB1*, *oeNtbZIP53*, and *oeNtMYB1 NtbZIP53* narcissus tissue relative to WT by quantitative RT–PCR. (A–F) Overexpression of *NtMYB1* and *NtbZIP53* in narcissus alters the expression of GA biosynthetic enzymes genes (*NtKS1*, *NtGA20ox*, *NtGA2ox*, *NtGA3ox*, and *NtGID1*). Different letters indicate statistical significance of values in *oeNtMYB1*-, *oeNtbZIP53*- or *oeNtMYB1*/*oeNtbZIP53*-transformed narcissus (*n* = 9) using Tukey’s test (*P* < 0.01). (G) Phenotype of *Arabidopsis atbzip53* mutant and *oeNtMYB1* or *oeNtbZIP53* in *atbzip53*-background complementation lines. Scale bar = 5 cm. (H) Diameter of rosette of *Arabidopsis atbzip53* and *oeNtMYB1* or *oeNtbZIP53* in *atbzip53*-background complementation lines. Different letters indicate statistical significance (*n* = 10).

Next, we functionally characterized the transcriptional regulatory activity of NtMYB1 and NtbZIP53 on the targeted genes in narcissus. For this purpose, we conducted a genome walking assay to clone the promoters of *NtGA2ox2*, *NtGA2ox8*, *NtGA3ox1*, and *NtGA3ox2* (downstream genes of NtMYB1); however, the promoter sequence of *NtGA3ox1* and *NtGA2ox2* could not successfully be cloned; a short promoter of *NtGA2ox2* was cloned and it has no function. Alternatively, *NtGA2ox8* is also co-expressed with *NtMYB1* and *NtbZIP53* (Fig. 5C). The promoter sequences were submitted to the NCBI database and analysed using the PlantCARE program (https://bioinformatics.psb.ugent.be/webtools/plantcare/html/) [25]. The analysis identified three MYB binding elements located −905, −1161 and − 1310 bp upstream of the *NtGA2ox8* coding sequence and two MYB binding elements −489 and − 291 bp upstream of *NtGA3ox2* (Supplementary Material Fig. S9). To determine whether NtMYB1 or NtbZIP53 or both directly bind to the promoters of *NtGA3ox2* or *NtGA2ox8* and regulate their transcription, we carried out a dual-luciferase reporter assay to measure the binding activity of NtMYB1 or NtbZIP53 or both with the promoters of *NtGA3ox2* or *NtGA2ox8* in tobacco leaf cells injected with the region containing many MYB motifs (CAACAG or GTTGTC) and the G-box (Fig. 6A and D). As illustrated in Fig. 6B and E, NtMYB1 repressed luciferase gene expression driven by *NtGA3ox2* or *AtGA2ox8* promoter; in contrast, NtbZIP53 activated luciferase gene expression driven by *NtGA3ox2* or *AtGA2ox8* promoter in fluorescence imaging in tobacco leaf. Furthermore, luciferase activity was assayed *in vivo* in the narcissus leaf protoplast system. Remarkably, the luciferase activity in narcissus protoplasts co-transfected with oeNtMYB1 and *NtGA3ox2* promoter was 4-fold lower than that in cells transfected with oeNtMYB1 and empty vector (negative control), while the luciferase activity in cells co-transfected with oeNtMYB1 and the *NtGA2ox8* promoter was 3-fold lower than that in cells transfected with oeNtMYB1 and empty vector (pGreen, *T7pro:LUC*), used as negative control (Fig. 6C and F). In contrast, the luciferase activity in narcissus protoplasts co-transfected with oeNtbZIP53 and *NtGA3ox2* promoter was 2-fold higher than that in cells transfected with oeNtbZIP53 and empty vector, while the luciferase activity in cells co-transfected with oeNtbZIP53 and the *NtGA2ox8* promoter was 8-fold higher than that in cells transfected with oeNtbZIP53 and empty vector (Fig. 6E). Surprisingly, the luciferase activity in narcissus protoplast cells co-transfected with oeNtMYB1/NtbZIP53 and *NtGA2ox8* promoter was 7-fold higher than that in cells transfected with oeNtMYB1/NtbZIP53 and empty vector. However, the luciferase activity in cells co-transfected with oeNtMYB1/NtbZIP53 and the *NtGA3ox2* promoter was 2-fold lower than that in cells transfected with oeNtMYB1/NtbZIP53 and empty vector (Fig. 6C and F). These data demonstrated that NtMYB1 or NtbZIP53 or both could specifically bind to the promoters of *NtGA3ox2* and *NtGA2ox8*, and that NtMYB1 repressed their expression and NtbZIP53 activated their expression, respectively. NtMYB1 interacting with NtbZIP53 enhanced the activation of NtbZIP53 on *NtGA2ox8* and the repression of NtMYB1 on *NtGA3ox2*.

**Figure 6:**
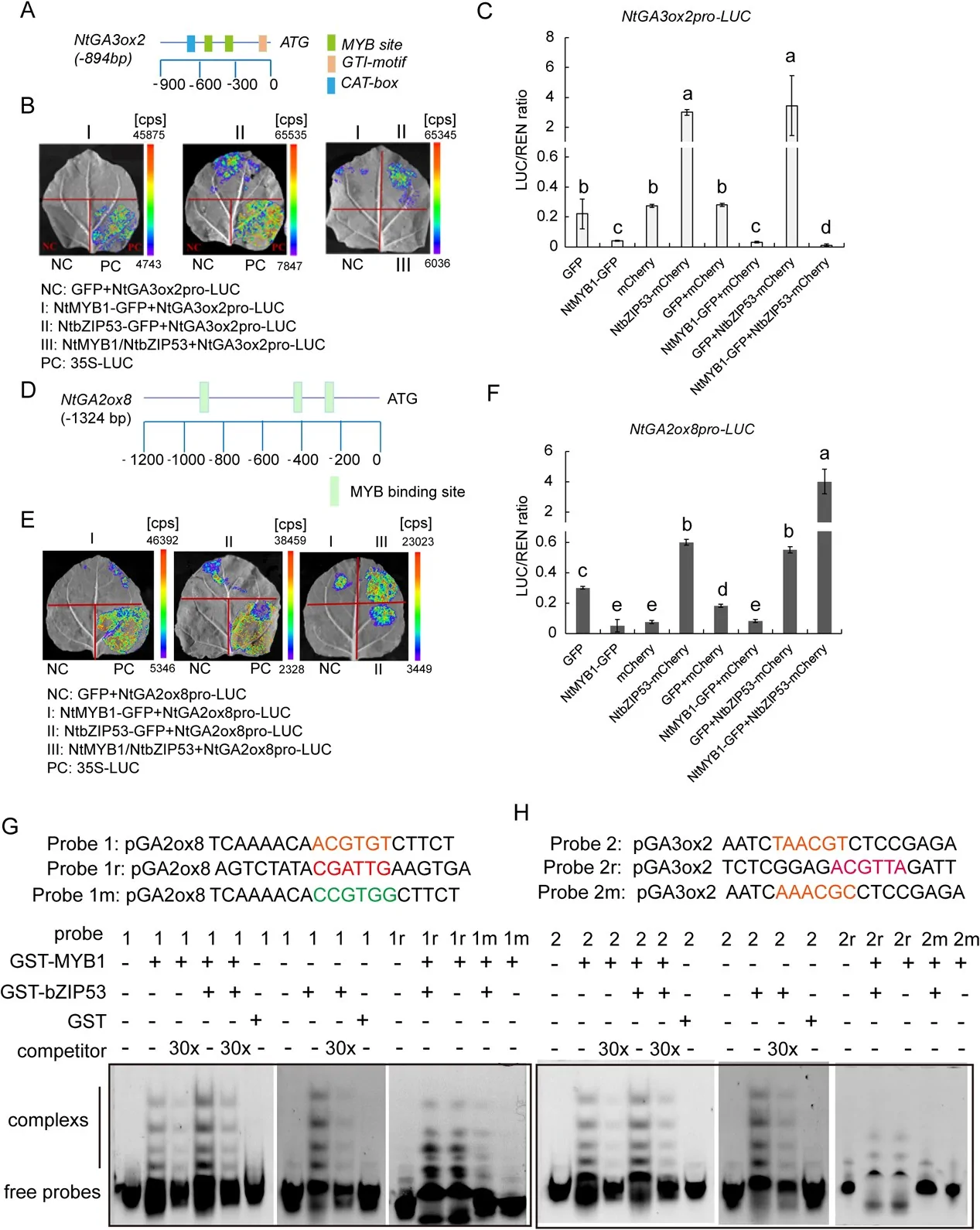
NtMYB1 and NtbZIP53 directly bound to the promoter region of *NtGA3ox2* and *NtGA2ox8* genes in tobacco leaf and narcissus protoplast using dual-luciferase and EMSA assays. (A) Promoter region of *NtGA3ox2*. (B) Dual-luciferase detection with imaging in tobacco leaf for oeNtMYB1or oeNtbZIP53 or both and *NtGA3ox2pro*. (C) Dual-luciferase detection with digital luciferase activity ratio (LUC/REN) in narcissus protoplasts for oeNtMYB1 or oeNtbZIP53 or both and *NtGA3ox2pro*. (D) Promoter region of *NtGA2ox8*. (E) Dual-luciferase detection with imaging for oeNtMYB1or oeNtbZIP53 or both and *NtGA2ox8pro*. (F) Dual-luciferase detection with digital luciferase activity ratio (LUC/REN) in narcissus protoplast for oeNtMYB1or oeNtbZIP53 or both and *NtGA2ox8pro*. Different letters indicate statistical significance of values for narcissus protoplasts co­transformed with LUC plasmids fused with the promoter of NtGA3ox or NtGA2ox8 and oeNtMYB1 or oeNtbZIP53 or both (*n* = 5 replicates) using Tukey’s multiple factor test: *P* < 0.05. (G, H). EMSA detection. Non-labeled probes were used as competitors (30×, 30-fold). 1r and 2r were reverse sequences; 1m and 2m were mutated sequences. Recombinant proteins GST-NtMYB1 and GST-NtbZIP53 purified from them expressed in *E. coli.*

Next, the recombinant NtMYB1 and NtbZIP53 proteins expressed in *Escherichia coli* were extracted and purifed with GST beads (Supplementary Material Fig. S10). The results of the EMSA assay showed that complex bands appeared when the reconbinant NtMYB1, NtbZIP53, or NtMYB1 plus NtBZIP53 (1:1) proteins incubated with the probes for the regions containing the binding sequence (probe 1: ACGTGT; probe 2:TAACGT), the reverse sequence (1r and 2r), and the mutation sequence (1m or 2m) of *NtGA3ox2* or *NtGA2ox8* promoters were labeled with Cy5; the intensity of complex bands (laddering bands) in the lane with NtMYB1 plus NtBZIP53 proteins was stronger than with NtMYB1 or NtbZIP53 alone, while the intensity of the complex bands was decreased when 30-fold non-labeled probes (cold competitor) were added before incubation, and when mutant sequence probes were used. This indicates that NtMYB1 and NtbZIP53 proteins specifically bound to the region of promoters of *NtGA3ox2* and *NtGA2ox8*, and the interaction of NtMYB1 and NtbZIP53 enhanced their binding affinity to the target promoters.

### NtMYB1 interacting with NtbZIP53 promotes GA_3_ accumulation and reduces GA_1_ content in narcissus

The soluble 2-oxoglutarate-dependent dioxygenases (GA_3_ox) have been reported to be involved in the formation of the bioactive GA_1_, GA_3_, and/or GA_4_. Both GA_3_-oxidase and GA_2_-oxidase have been considered as important regulators of the biosynthetic pathway in *Arabidopsis* [11–13]. Next, transgenic narcissus plants with *oeNtbZIP53* and *oeNtMYB1/NtbZIP53* were generated (Supplementary Material Fig. S8). Overexpression of *NtMYB1* or *NtbZIP53* or *oeNtMYB1/NtbZIP53* in narcissus led to a tiny-bulb and less branching bulb phenotype compared with WT (Fig. 7A and B). The phenotypes of *Arabidopsis* overexpressing *NtMYB1*, *NtbZIP53*, and *NtMYB1/NtbZIP53* showed a similar tiny plant, but with a more branching phenotype (Supplementary Material Fig. S11). Furthermore, in the *oeNtMYB1*, *oeNtbZIP53*, and *oeNtMYB1/NtbZIP53* transgenic narcissus and *Arabidopsis*, the contents of various GA species, including GA_1_, GA_3_, GA_20_, and GA_5_, were measured, and the results showed that the content of GA_1_ was significantly reduced in both *oeNtMYB1* and *oeNtMYB1*/*NtbZIP53* transgenic narcissus tissue, while the content of GA_3_ was significantly increased in both *oeNtMYB1* and *oeNtMYB1*/*NtbZIP53* transgenic narcissus tissue and *Arabidopsis* leaves (Supplementary Material Fig. S11). The amounts of GA_20_ and GA_5_ in narcissus were increased in both *oeNtbZIP53* and *oeNtMYB1*/*bZIP53* narcissus tissue (Fig. 7C and D). Thus, it is supposed that NtMYB1 or NtbZIP53 or both can specifically bind to the promoters of *NtGA2ox8* and *NtGA3ox2*, that NtMYB1 repressed their expression and NtbZIP53 activated their expression; oeNtMYB1 recruiting NtbZIP53 enhanced the activation of NtbZIP53 on *NtGA2ox8*, resulting in conversion of GA_1_ to the non-active species GA_8_ and enhancing the repression of NtMYB1 on *NtGA3ox2*, resulting in GA_20_ accumulation, then conversion of GA_5_ to GA_3_.

**Figure 7:**
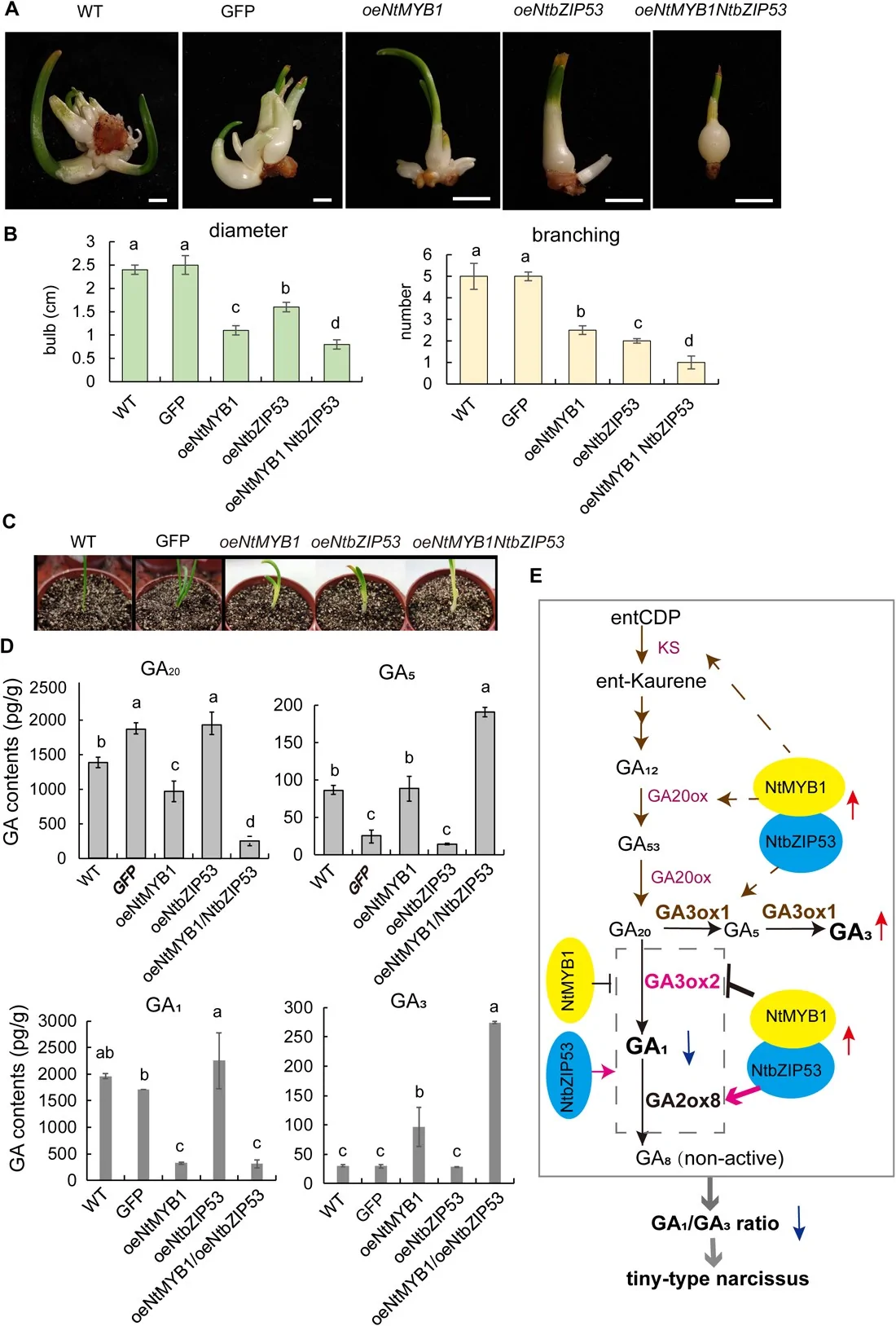
Phenotypes and GA contents in *oeNtMYB1*, *oeNtbZIP53*, and *oeNtMYB1 NtbZIP52* narcissus tissues. (A) Phenotypes of *oeNtMYB1*, *oeNtbZIP53*, and *oeNtMYB1*/*NtbZIP53* narcissus compared with *oeGFP* and WT narcissus. (B) Bulb diameter and branching number in *oeNtMYB1*, *oeNtbZIP53*, and *oeNtMYB1*/*NtbZIP53* narcissus compared with *oeGFP* and WT narcissus. (C) Leaf of *oeNtMYB1*, *oeNtbZIP53*, and *oeNtMYB1*/*NtbZIP53*, *oeGFP*, and WT narcissus. (D) Contents of GAs in *oeNtMYB1*, *oeNtbZIP53*, and *oeNtMYB1/NtbZIP53* narcissus compared with *oeGFP* and WT narcissus. Statistical analysis was by Tukey’s test; different letters indicate significance (*P* < 0.05) (*n* = 5; B, D). (E) Hypothetical model of NtMYB1 recruiting NtbZIP53 and regulating GA_1_ and GA_3_ biosynthesis, leading to a tiny-type plant.

## Discussion

The GA catabolic gene *GA2ox1* and the GA biosynthetic gene *OsGA3ox2* were expressed with tissue and cell-specific expression patterns of biosynthesis or deactivating enzymes, which can be used for the fine tuning of plant growth [26]. Therefore, as changes in the contents of diverse GAs cause a mix of positive and negative effects on traits of agronomic importance (e.g. reduced plant size—a mini architecture, reduced seed yield, promoting flowering), it is of utmost importance to determine how one GA effect could be uncoupled from another [7]. In this study, we identified an *NtMYB1* and an *NtbZIP53* gene in narcissus. Overexpression of *NtMYB1* or *NtbZIP53* in transgenic lines of *Arabidopsis* and narcissus led to upregulation of the gene expression of upstream biosynthetic enzymes of the GA biosynthesis pathway, while oeNtMYB1 or oeNtbZIP53 antagonistically controlled *GA3ox2* and *GA2ox8* transcription, and NtMYB1 recruiting NtbZIP53 enhanced the repression of *GA3ox2* by NtMYB1 and the activation of *GA2ox8* by NtbZIP53, resulting in reduced active GA_1_ content and increased GA_3_ content, leading to a tiny plant type. This study suggested the potential role of NtMYB1 as a repressor of *GA3ox2* to inhibit GA_1_ biosynthesis and of NtbZIP53 as an activator of *GA2ox8* for conversion of active GA_1_ to inactive GA_8_. NtMYB1 interaction with NtbZIP53 significantly reduced GA_1_ biosynthesis and promoted GA_3_ biosynthesis in narcissus and *Arabidopsis* (Fig. 7E).

### NtMYB1 and NtbZIP53 co-suppress the GA_1_/GA_3_ ratio, leading to a tiny plant type in both *Arabidopsis* and *Narcissus*

The soluble 2-oxoglutarate-dependent dioxygenases, GA3oxs, have been reported to be involved in the formation of bioactive GA_1_, GA_3_, and/or GA_4_. Both GA_3_-oxidase and GA_2_-oxidase have been considered to be important regulators of the biosynthetic pathway in biochemical and molecular studies in *Arabidopsis* [11–13]. In transgenic *oeNtMYB1 Arabidopsis* and narcissus plants, the ratio of GA_1_ to GA_3_ was reduced, while with NtMYB1 recruitment of NtbZIP53 the ratio of GA_1_ to GA_3_ was significantly reduced. Although the amount of GA_3_ in leaves is much lower than that of GA_1_, overexpressing *oeNtMYB1* and *oeNtMYB1*/*NtbZIP53* increased the amount of GA_3_, and the tiny phenotype occurred in *oeNtMYB1*, *oeNtbZIP53*, and *oeNtMYB1*/*NtbZIP53 Arabidopsis* and narcissus. It seems that the GA_1_/GA_3_ ratio is essential for controlling plant rosette size. It has reported that in *Arabidopsis* GA_4_ and GA_1_ accumulate in growing tissue, flower buds, flowers, and the early developing silique, and in mid-seed development [27–29]. In this study, GA_4_ was undetectable in *Arabidopsis* and narcissus leaves, but could be detected in the roots of narcissus, but the GA_4_ level did not alter in various overexpression lines, suggesting that NtGA3ox2 might not function from GA_9_ to GA_4_ (Fig. 4B); exogenous application of GA_3_ did not recover the short root phenotype of *oeNtMYB1 Arabidopsis*; and GA_3_ favors stem elongation through cell growth regulation and in response to environmental stress and accumulates in the growing stem. GA_3_ application promotes the transcription of xyloglucan endotransglycosylase (XET), α- and β-expansins [30] and reduces the expression of PHYTOCHROME-INTERACTING FACTOR 4 (PIF4) under a short-day photoperiod [51]. In blueberry, the inflorescence number and vegetative growth increased with GA_3_ treatment [31]. However, in *Matthiola incana*, GA induced stem elongation but no effect on flowering was observed [32]. These results highlight diverse aspects of GAs species, as the species-specific responses and the variability found in some cases can discourage their application. In both *Arabidopsis* and narcissus, NtMYB1 and NtbZIP53 seem to have conserved function for plant size, though not for branching. NtMYB1 seems to dominantly inhibit GA_1_ biosynthesis via *NtGA3ox2* compared with NtbZIP53, and activated GA_3_ biosynthesis via redirection of accumulated precursors towards GA_3_ or *NtGA3ox1*, indirectly affected by NtbZIP53. The results also do not exclude NtbZIP53 activating NtGA2ox8, resulting in feedback regulation, which needs further biochemical evidence.

### NtMYB1 and NtbZIP53 control expression of various targeted genes such as *NtGA3ox* or *NtGA2ox* or *NtNCED1*/*2* at the transcriptional level in a developmental-dependent manner

A link between pigment metabolites and hormones can be established due to their biochemical origin and similar biological roles. Three branches of the methylerythritol phosphate pathway involved in biosynthesis of carotenoids (also ABA), biosynthesis of the phytol side chain of chlorophylls, and diterpenoid biosynthesis have been identified in many plants [33–37]. In this case, Geranylgeranyl pyrophosphate (GGPP) is not only used for carotenoid biosynthesis, but also serves as an immediate substrate for biosynthesis of the phytol side chain of chlorophylls and of diterpenoids such as gibberellins. Allocation of GGPP among different downstream metabolic branches largely determines the biosynthetic capability of each branch [33]. This can explain why, in *Arabidopsis* overexpressing *NtMYB1* and *NtbZIP53*, high levels of carotenoids and chlorophyll, as well as a low content of GA_1_, were detected (Figs 1 and 7) in the seedling stage, suggesting the presence of competition of carotenoids or the phytol side chain of chlorophylls and terpene backbone biosynthetic branches for GGPP [23]. In a previous study, we showed that NtMYB1 directly targets the MYB motif region of *NtNCED1* and *NtNCED2* promoters and activates their transcription [52]. NCED protein is a key rate-limiting enzyme for the conversion of violaxanthin to xanthoxin, which is responsible for abscisic acid biosynthesis [38, 39]. Collectively, an NtMYB1–NtNCED1/2 module plays a crucial role in controlling ABA biosynthesis and the accumulation of β-carotene and ABA. Strikingly, *NtMYB1* overexpression led to a decrease in the level of GA_1_ and an increase in chlorophyll in 3-week-old seedlings (Supplementary Material Fig. S1), while causing tepal senescence and leaf yellowing in both narcissus and *Arabidopsis* after the bolting stage (8 weeks old) [52]. This may be because NtMYB1 or NtbZIP53 targets and represses or activates antagonistically the transcription of *NtGA3ox2* and *NtGA2ox8*, respectively, at an early developmental stage. High or low transcript levels of *NtGA2ox8* or *NtGA3ox2* are shown in a transcriptome dataset (Supplementary Material Fig. S12). A second possibility is that currently a small amount of evidence has shown that the promoter regions of *NCED* and *GA3ox* or *GA2ox* are modified by epigenetic marks during leaf senescence, resulting in alteration of the metabolic pathway, in a developmental-dependent manner. For example, the histone acetylation of the *NCED* gene by HDA19 contributes to positive regulation of ABA synthesis in *Arabidopsis* and by HDA15 in rose petal senescence [40–42]. Several GA_2_ox enzymes orchestrate the catabolism of GAs, in which GA_2_ oxidases (GA_2_oxs) were notably regulated epigenetically by HDACs in *Arabidopsis* root [43, 44]. Furthermore, *NtMYB1* and *NtbZIP53* gene bodies contain the EAR motif and ZIP motif, respectively. The EAR motif has the potential to be involved in epigenetic (histone acetylation) regulation of transcriptional repression. bZIP has a consensus binding sequence of 5′-TTG-CG-CAA-3′, with a central CpG dinucleotide flanked by two CpA sites. Methylation of the Cp(G/A) sites produces a DNA motif, with every pyrimidine having a methyl group at the ring carbon C5 position of thymine or 5mC [45, 46]. These scientific insights into whether the epigenetic modification/regulation of key enzymes regulates the metabolic flux direction of the ABA biosynthetic pathway and the GA biosynthetic pathway in narcissus to regulate the production of various GA species will be challenged in future studies.

## Conclusions

Overexpression of *NtMYB1*/*NtbZIP53* transgenic lines in *Arabidopsis* and narcissus upregulated the gene expression of upstream biosynthetic enzymes of the GA biosynthesis pathway, while *oeNtMYB1* or *oeNtbZIP53* antagonistically controlled *GA3ox2* and *GA2ox8* transcription. NtMYB1 recruitment of NtbZIP53 enhanced the repression of *GA3ox2* by NtMYB1 and the activation of *GA2ox8* by NtbZIP53, resulting in reduced active GA_1_ content and increased GA_3_ content, leading to a tiny plant type. This provides a conceptual basis for controlling the architecture of Chinese narcissus.

## Materials and methods

### Sample collection

*Narcissus tazetta* cv. ‘Jinzhanyintai’ was purchased from a local farmer (Zhangzhou, Fujian, China). After the old bulb scales and roots were removed, cleaned bulbs were sand-cultured in a greenhouse set at 23°C with constant illumination (16 h light/8 h dark) [22]. Narcissus tepals (combined organ of petal and calyx) and corona were first collected at the fifth day (samples T1 and C1) when sheathed leaves emerged from the bulbs, and then collected at three other stages (samples T2–T4 and C2–C4) as the umbel in the spathe developed. All samples were immediately frozen in liquid nitrogen and stored at −80°C. This material was used to produce the transcriptome sequencing data of Yang *et al*. [23] and the *NtMYB1* and *NtbZIP53* expression profile.

### Transformation and treatment of narcissus plants

Transgenic bulbs of *N. tazetta* were generated with a stable genetic transformation system for *Narcissus* callus (patent CN202210162166.8). In short, *Narcissus* ovaries in the S0–S1 period were used as explants to induce callus after disinfection, and *Agrobacterium* EHA105 carrying the NtMYB1or NtbZIP53 overexpression vector (*35Spro:NtMYB1-GFP-GUS* or *35Spro:NtbZIP53-GFP-GUS* or both) was used to transform narcissus callus following 3 d of co-cultivation. After 27 d of screening in a medium containing 25 μg ml^−1^ kanamycin, the transformed callus was used for material identification. The positive callus was continually induced to generate transgenic plants. The *T*_1_ generation plants were used to collect bulb and root material.

*Arabidopsis* (Columbia) was transformed with an *NtbZIP53*-overexpressing plasmid (*35Spro:NtbZIP53-GFP-GUS*) by using a floral dip assay. Six-week-old *oeNtMYB1 Arabidopsis* plants and *T*_1_ transformed narcissus bulbs were treated with 100 μM GA_3_ (Sangon Biotech (Shanghai, China) for further assays and *T*_1_ transformed narcissus bulbs were treated by adding 100 μM GA_3_ to the medium.

Tobacco (*Nicotiana benthamiana*) plants were cultivated in a long-day climate chamber. The long-day cultivation conditions were set as follows: a photoperiod of 16 h light/8 h dark, a constant temperature of 23°C, and a humidity of 60%.

### Genome walking assay

Genomic DNA was isolated from leaves of *N. tazetta* at the S2 stage. The 5′ upstream sequences of the *NtGA3ox2* and *NtGA2ox8* coding sequences were cloned using Takara’s Genome Walking Kit (cat. no. 6108), and the primer sequences are shown in Supplementary Material Table S1.

### Plasmid construction

The *oeNtMYB1* plasmid was provided by previous work and the *oeNtbZIP53* plasmid was constructed in the same way as described [52]. Briefly, pK7FWG2 vector was used as the backbone to construct the plasmids *35Spro:NtMYB1-GFP-GUS* and *35Spro:NtbZIP53-GFP-GUS*. Plasmid *pCBIM-GFP-GUS* (*35Spro:GFP*) was used as a positive control in narcissus protoplast transformation experiments. pK7FWG2 (*35Spro:GFP*) or modified *pCAMBIA3301* maize polyubiquitin promoter (*ZmUbipro:GFP*) instead of *Arabidopsis ACT2* promoter and referred to as *ACT2pro:GFP* was used to construct the overexpression vectors in a dual-luciferase assay in tobacco leaf. The dual luciferase vector under the control of the *T7* promoter was constructed using pGreen (*T7pro:LUC*). In the dual-luciferase reporter system, the *Renilla* luciferase gene (*REN*) under the control of the NOS promoter was used as an internal control.

### Bimolecular fluorescence complementation assay

The *YFPc-NtMYB1*, *YFPn-NtbZIP53*, *YFPn-NtGRAS*, *YFPn-NtMYB1*, *YFPc-NtbZIP53*, and *YFPc-NtGRAS* plasmids were extracted using the EndoFree Plasmid Midi Kit (CW2105), and were then co-transformed into epidermal cells of tobacco leaf mesophyll and fluorescence was observed under a laser confocal microscope (Leica SP8) after 16 h. Co-transfection of YFPc and YFPn empty vector was used as the negative control.

### Co-immunoprecipitation and immunoblotting assays

The combination of constructs expressing the corresponding MYB1-HA-tagged proteins and NtbZIP53-GFP tagged proteins were co-transformed into narcissus protoplasts and co-injected into tobacco leaf. After incubation for 16 h, the transfected protoplasts were harvested for co-immunoprecipitation (Co-IP) and immunoblotting assays as described previously [47]. The HA-tagged proteins were immunoprecipitated with anti-GFP antibody-coupled beads (TransGen, HT801, 1:1000 dilution). The immunoprecipitated proteins were then separated by 13.5% SDS–PAGE, blotted and analysed by immunoblotting with anti-HA (anti-HA affinity matrix, Roche) and anti-HA-peroxidase (Roche, 3F10, 1:500 dilution) antibodies.

### Measurement of various gibberellins

Leaves from *Arabidopsis* or bulb and root from *T*_1_ plants of *N. tazetta*, transformed bulbs, and *T*_1_ transgenic *Arabidopsis* plants were collected, and the contents of pigments and hormones were measured by HPLC [53]. Briefly, ~100 mg of freeze-dried bulb and roots of narcissus or *Arabidopsis* leaves were homogenized with 1 ml of a methanol–dimethyl sulfoxide mix (1:1, v/v) at 40°C for 30 min with agitation (190 rpm) in darkness. A final volume of 10 μl of homogenate was used for injection into the HPLC instrument. The following reagents were purchased: standards GA_12_, GA_53_, GA_20_, GA_5_, GA_3_, GA_4_, and GA_1_ from Sigma– Aldrich (St Louis, MO, USA). All standards were prepared with methanol–dimethyl sulfoxide (1:1, v/v) and stored at −20°C before use.

### Dual-luciferase assay

The dual-luciferase experiment followed the method of Rodriguez-Concepción *et al*. [48], with slight changes. The constructed *ACT2pro:NtMYB1-GFP* and *ACT2pro:NtbZIP53-mCherry* overexpression vector (as effector), empty: LUC, *NtGA3ox2pro:LUC*, *NtGA2ox8pro:LUC* vector (as reporter), GFP-tagged empty vector (as negative control for effector) and pGreen (*35S:LUC*) vector (as positive reporter) were transferred into *Agrobacterium* GV3101 (pSoupp19) competent cells, and the positive clones were screened and identified, and the *Agrobacterium* resuspended solution with various combinations of effector and reporter was mixed in an equal ratio of 1:1 and used for tobacco injection and transforming the protoplasts from narcissus leaves. The experimental group (e.g. *ACT2pro:NtMYB1-GFP* + *NtGA3ox2pro:LUC; ACT2pro:NtMYB1-GFP/NtbZIP53-mCherry + NtGA3ox2pro:LUC; ACT2pro:NtbZIP53-mCherry + NtGA3ox2pro:LUC*), the negative control (*ACT2pro:NtMYB1-GFP* + empty LUC report vector), and the positive control group (pGreen) were injected into the same leaf. The injected tobacco plants were cultured in the dark for 12–24 h. After 2–3 d, the GFP and mCherry fluorescence signals were observed under a fluorescence microscope or a laser confocal microscope (Leica SP8) to illustrate the expression of effector protein.

The luciferase activity of each sample was qualitatively analysed using an *in vivo* plant imaging system (Immunization Center of Fujian Agriculture and Forestry University). Subsequently, the relative luciferase activity was quantified with a microplate reader [49]. The ratio of firefly to *Renilla* luciferase activity (LUC/REN) was calculated as the relative luciferase activity.

### Reverse transcription–quantitative PCR

Total RNA was extracted from bulbs of *N. tazetta* following the protocol of Ren *et al*. [22]. Total RNA was extracted from leaves using TRIzol extraction reagent (Thermo Fisher Scientific), according to the manufacturer’s protocols. The primer sequences used for expression analysis of the candidate genes are shown in Supplementary Material Table S1. The endogenous *AtGAPC2* and *NtACTIN* were used as the reference genes. The data for *NtACTIN* are shown in the figures. Relative expression levels of target genes were calculated using the 2^-ΔΔCt^ method of Schmittgen and Livak [50]. In this experiment three biological replicates were tested, and each biological replicate contained leaves or tepals from three independent plants.

### Weighted gene co-expression network analysis

WGCNA is a common method for constructing gene co-expression networks [24]. WGCNA analysis of GA metabolism enzyme and related transcription factors was according to the description by Yang *et al*. [23].

### Statistical analysis

All experiments were performed using at least three biological replicates, and each measurement was repeated three times, as indicated in the legends. Duncan’s new multiple range test in one-way ANOVA was used to determine the significance of differences between treatments.

## Supplementary Material

Web_Material_uhag234

## Data Availability

All data are provided in the supplementary material. The plant materials will be provided by the corresponding author.
