# Supplementary material for: NtMYB1 and NtbZIP53 antagonistically regulate NtGA3ox2 and NtGA2ox8 mediating diverse GA biosyntheses and tiny-plant in Narcissus tazetta
Source: Hortic Res. 2026 Jun 8;13(11):uhag234. doi: 10.1093/hr/uhag234 (PMC13723081; doi:10.1093/hr/uhag234)
Supplement: Supplementary file 1 [file web_material_uhag234.zip › Supplementary Figs1-s12-tables1-r1 .pdf]

Supplementary information

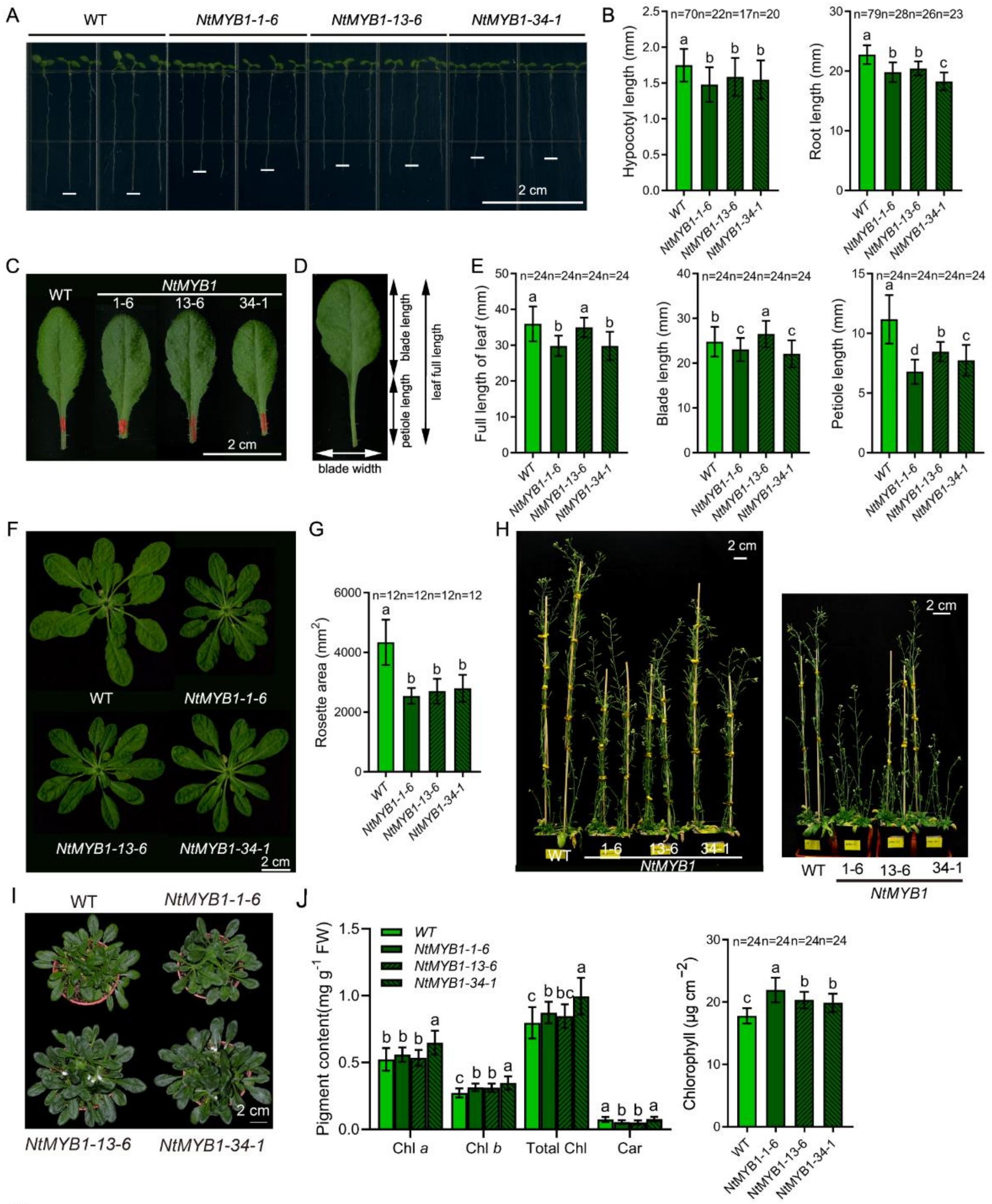

Figure S1 The " tiny " phenotype analysis of *oeNtMYB1* transgenic Arabidopsis

(A~B) Hypocotyl and root phenotypes and lengths of 7-day-old WT and *oeNtMYB1* transgenic Arabidopsis seedlings grown on 1/2 MS medium, error bars represent mean ± SD (n⩾17); (C~E) The blade length, petiole length and leaf full length of the leaf 11th of 36-day-old Arabidopsis rosette leaf were measured and analyzed; (F~G) Rosette area were analysis of 44-day-old plants; (H) *oeNtMYB1* transgenic Arabidopsis showed slender flower stem and more branching phenotype. (I) Phenotypic comparison of WT and *oeNtMYB1* at 3-week-old seedlings, including the dark green leaves; (J) chlorophyll content of the leaf 5th of 31-day-old Arabidopsis rosette. Statistical analysis is done by Turkey's test, different letter indicates significant (P<0.05).

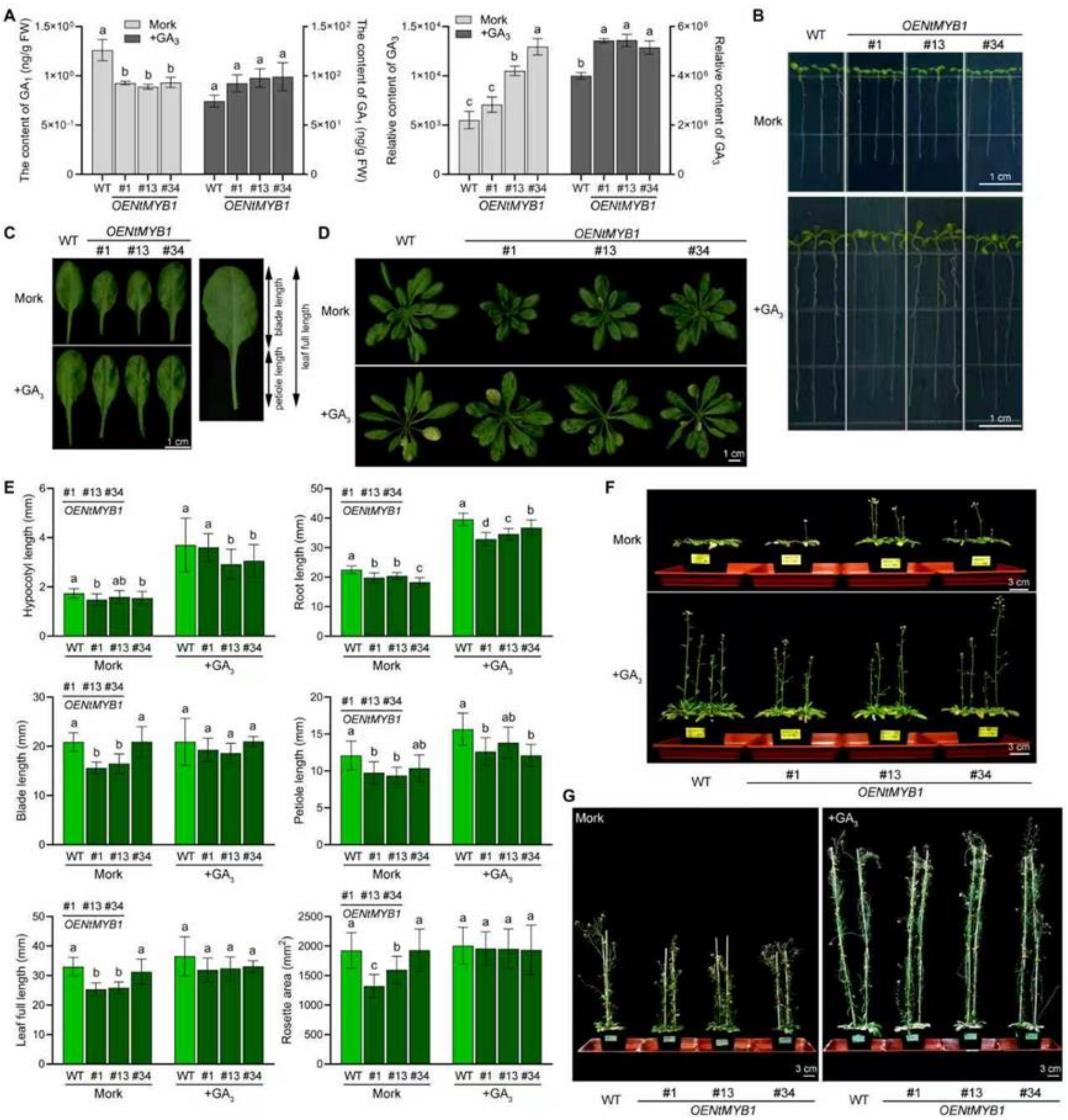

Figure S2 Exogenous gibberellin (GA3) application partially recscues the dwarfismic phenotypes of *oeNtMYB1* transgenic Arabidopsis.

(A) Endogenous GA_1 and GA_3 levels in rosette leaves of 37-day-old WT and transgenic plants treated with 100 μM GA_3 or mock. (B, E) Hypocotyl and root phenotypes (B) and quantification (mean ± SD, E) of 7-day-old seedlings grown on 1/2 MS with 10 μM GA_3 or mock. (C–E) Seventh leaf and rosette area traits (C, D) and quantification (mean ± SD, E) of 37-day-old plants. (F) GA_3 abolished early bolting and flowering in 40-day-old *OENtMYB1* plants. (G) GA_3 restored plant height of 8-week-old *OENtMYB1* to WT levels. One-way ANOVA with Tukey's test; different letters indicate $P < 0.05$.

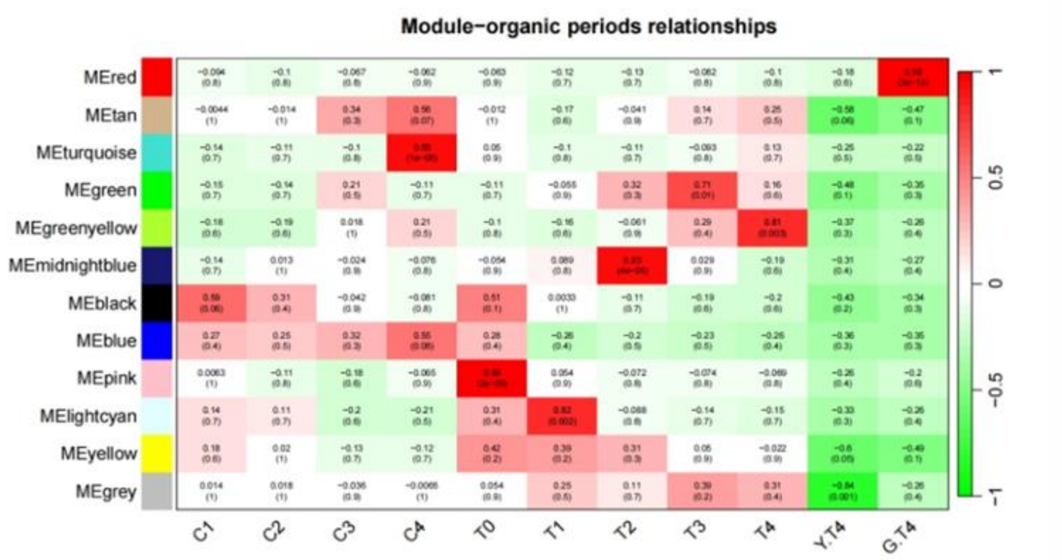

Figure S3 WGCNA analysis of the weighted gene co-expression network of TFs in the Chinese daffodil 'Jin Zhan Yin Tai'. The module-tissue period correlation and the corresponding P value (in parentheses) are shown on the left, representing twelve modules, while the color scale on the right indicates the module-trait correlation ranging from -1 (blue) to 1 (red). The parameter setup as 为 power = 14, minModuleSize = 30, and MEDissThres = 0.25.

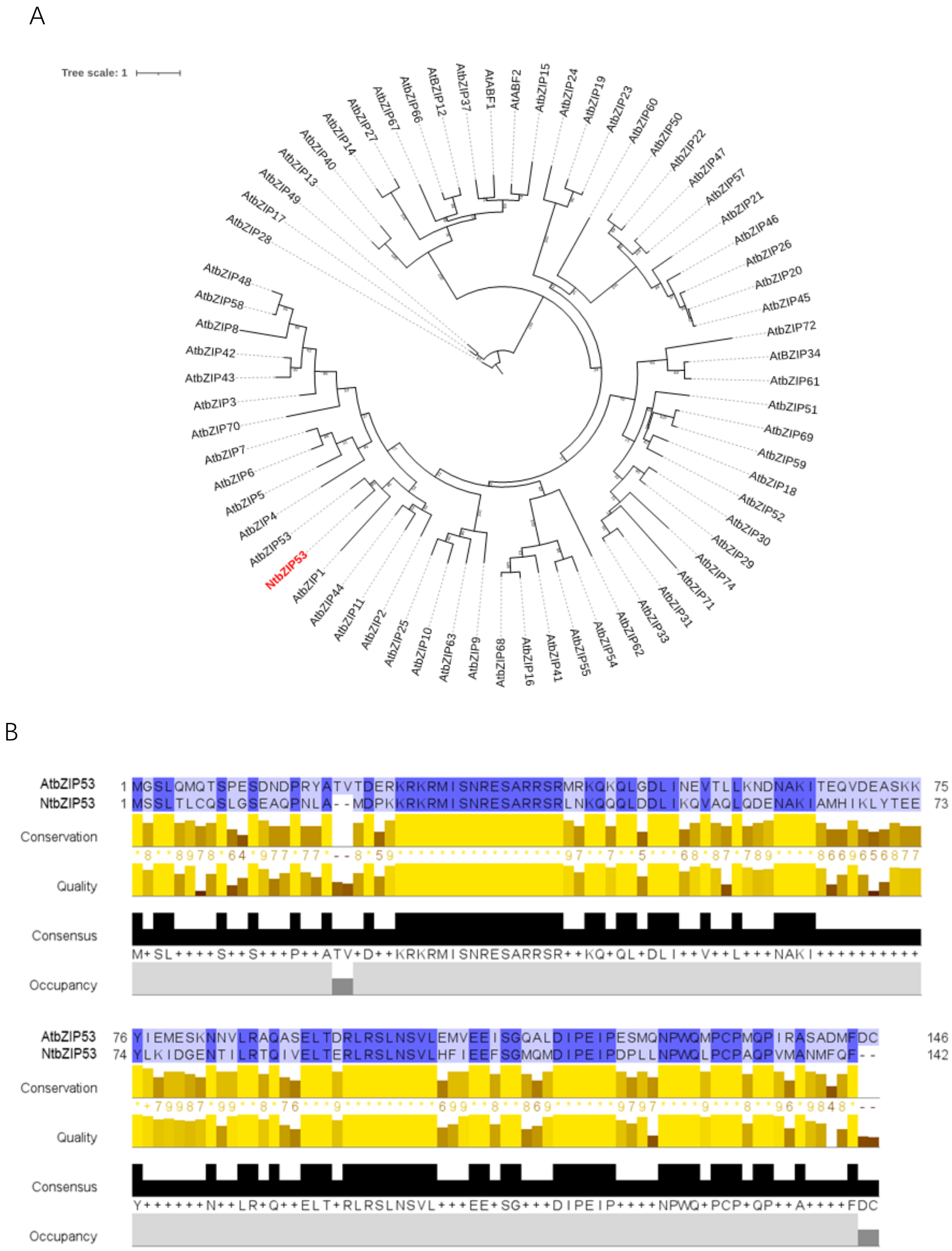

Figure S4 Phylogenetic tree of *NtbZIP53* with *AtbZIPs* (A) and comparison of protein sequences between NtbZIP53 and AtbZIP53 (B).

A

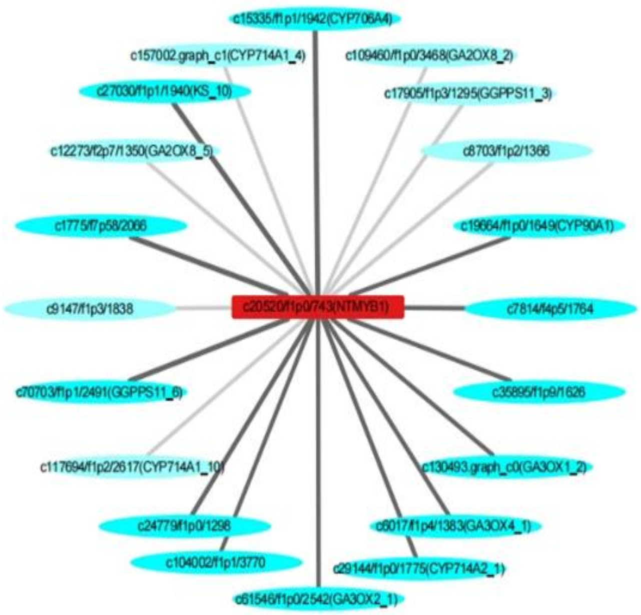

B

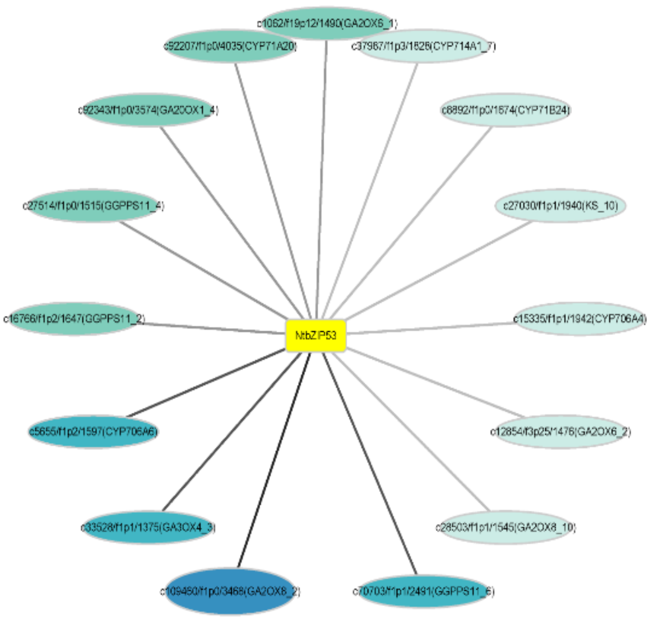

Figure S5 Coexpression network of *NtMYB1* (A) or *NtbZIP5*3 (B) with GA metabolic enzyme genes.

A correlation map of the top 10 correlated genes with the expression level of *NtMYB1* and the gene expression level of GA metabolism enzyme. The deeper the color of the lines, the stronger the correlation.

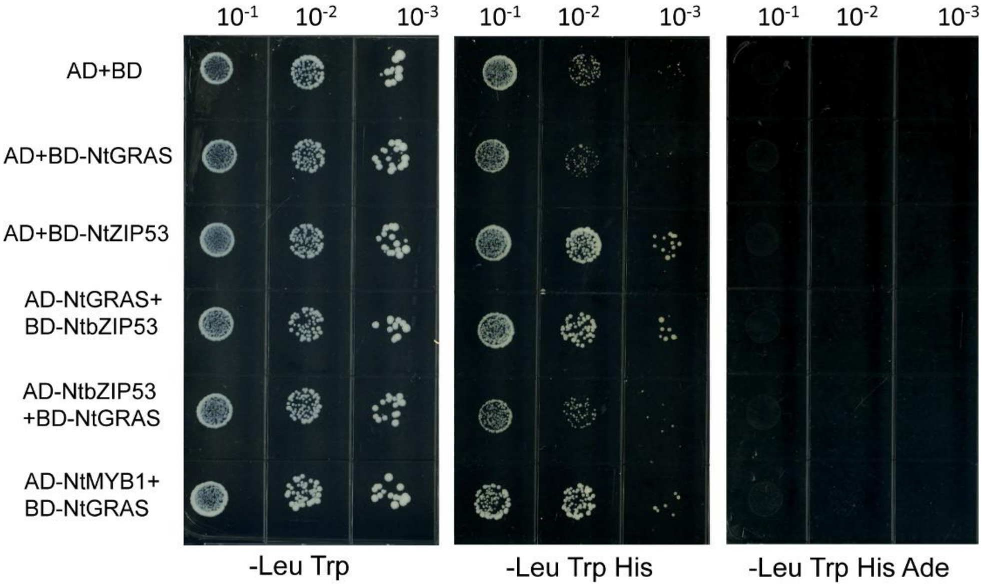

Figure S6 NtMYB1 or NtbZIP53 cannot interact with NtGRAS in yeast cells.

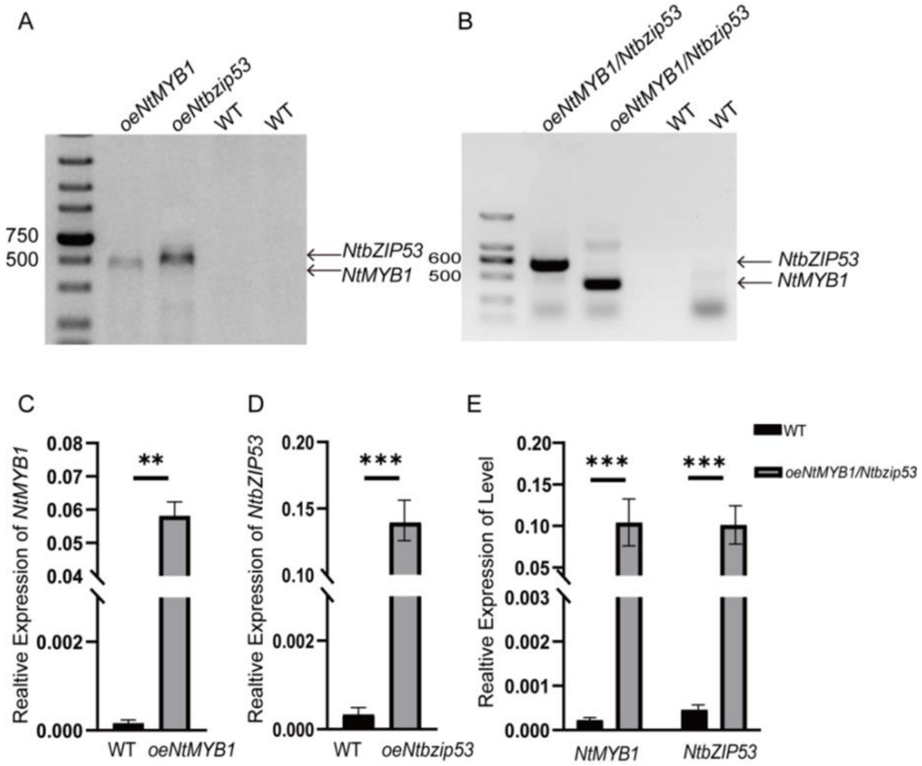

Figure S7 Identification of T1 generation materials of *Arabidopsis thaliana OENtMYB1* and *OENtbZIP53-OENtMYB1*. (A) Identification of Genomic DNA level of *OENtbZIP53 Arabidopsis thaliana*. (B)Identification of Genomic DNA level of *OENtbZIP53-OENtMYB1 Arabidopsis thaliana*. (C)The expression of *NtbZIP53* gene in *OENtbZIP53 Arabidopsis thaliana* was detected by RT-qPCR. (D)The expression of *NtbZIP53* gene in *OENtbZIP53-OENtMYB1 Arabidopsis thaliana* was detected by RT-qPCR. *AtGAPC2* was used internal control, 3 biological replicates ($p<0.001$).

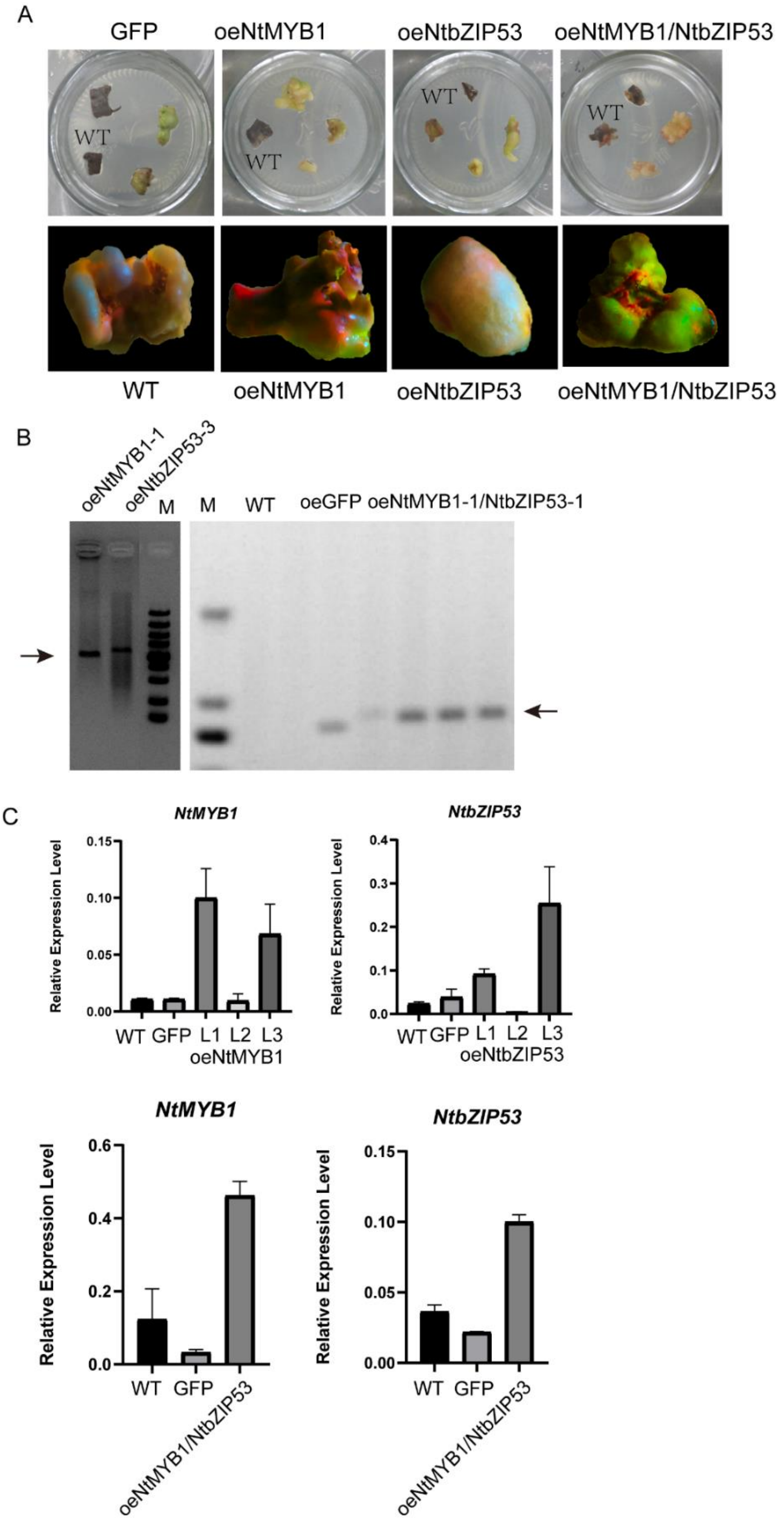

Figure S8 Identification of *oeNtMYB1*, *oebZIP53* and *oeNtMYB1/NtbZIP53* in transgenic narcissus. (A) Transforms of oeNtMYB1, oebZIP53 and oeNtMYB1/NtbZIP53 in narcissus tissues; (B) The detection of genome insertion by genomic DNA-PCR; (C) the transcript level of *oeNtMYB1* and *oebZIP53* in the *oeNtMYB1*, *oebZIP53* and *oeNtMYB1/NtbZIP53* transgenic narcissus tissues.

A.

The promoter sequence of *NtGA3ox2*

AGGAGGCAGGCCATATGGTACTAATAAAGCTTAATTTTTCATATTCCATAATTTGCCAAAGA
CTTTTGTGGGTCATGGGATTCATGGGCATCCATCCACGAGTGCTGTTCTCCAATATCAATC
TACCTAGACTATTATTCCAACTTTACTTCCTGATTTCCCTGTTCTTCCAACTCCTATAAATAC
CCCTCCTTATAATCACCCAAGCACATAGCGCAACTCACAAAGCTAAACAAGTTGATTAGAG
AGAGAGTAGTTTTTGGATAAATTTTGCAATGGATTCTTTCCCAGTTATCAACATGGAGAAGC
TTAAAGGCGAGGGGAAGGGTGCCGCGATGGATGTTCTTCAAGATGCTTGCGAGAACTGG
GGCTTTTTCGAGGTAATTATGCCAGATTATACCAGTGGATTATCTCACTTTTGCTTTTTATTT
ATTTAATCTGACAAGTACGGTTCTTACTAGATTTTGAACCATGGGATCACCCATGAGCTGTT
GGACAAGGTGGAAAGAATGACCAAGGATCACTACAAGAAATGCATGGAGGAAAGGTTCA
AGGAGTTTGCGAGCCAAGCACTCGAAAAGGGCGAAGAACTGGACAGGAGCAACATGGA
CTGGGAGAGCACATTCTTCTTGCGCCATCTTTCCGAATCTAACGTCTCCGAGATCCCCGA
TCTCGATGAAGAATACAAGTAATTAACAATTACATCAGGATATATAAAAATACACTCTCAATC
CTCCTCATAATAAAAATTATCACCCTCATGATCCACCCTT**ATG**

B.

The promoter sequence of *NtGA2ox8*

TTTTGATAATTTTGTCCTAGTCAAGAAGGTTGGGAATAAAAACAGTTATATTATTGTCATAT
GAATAAAGTCTTGGGCGGGTGGGGAGCATCCTTTATTTTTTTTTTTGAAAATGAAAAAAA
AATAAAGGGTGGTCCCCACCCGCCGAAGACTTTATTCACAGCTATGATGGCTGTGAATA
AAATCAGTTACATTATTCTCATATATAAACACACACACTAATTTCATTATAGTGTAATTAAT
TAAAATGTAAGTGGATTCAAATATTTCCAAAATCAAGTAGTTTCCATTGAATTTGAATGCTA
TTAATAATCTCAGTCCATTTAAATATTGGATCAATAGATACATGCAAACGATGCAATGTCT
GTTCCATATTCACTCAGTTTATACCTTGGAGATATCTGTCATCAGAAAGCGAGTTGTTGG
AGAAGTCACTTCAATCGTATAGACTCGGTCGAATCCGATACTTTGGAGTAATTGTCATAG
AAGTAGACCAACTTTAGTTTCATTCAAGGTCTATCTGGGTGCAAATTTAAGATTTTTTTAG
TTGATCTGCCTCTATGAAGGTATACCGATAAAATTTCCTCCGGCCAAAGAGAGGTCTTAG
ATACAAATAACTTGCTACTTATTGTAATATGAAATATCAACTGAACTTTTAAACTATTTTCT
CTCAATTTTTCTTCAAGTTGTTCCGAGACTGGGACTTCTAGTGTAATGCACCGATACATAC
AACAACTTAGATAATCCTCACTGAAATTGCCCCTGGGTCCTCCTGTTTCAATCATAGAGT
AAGAAGCACATGCATTGACCACATGACATGTTTACATACATGCATGAACATGCATTGACC
ACATTCCTCATGATCCAGTGTCGGCTAAAAATGAAATAACAGAATGTGCATATGTTTTTCA
TTTTCCAGAAGTATTTTAATTTTTGTGAGATAAATTGGCCTTAATCATACGTTTGGAAATTT
GCAAACAAATGTTAGCGAAATCTGCCCTCCCCGAAACCACTTCCCAATCATACTGTGGTC
GGTGGTCATCCTGCACCTGACTCTGCATTCTACCATTCAATTAGAAGACACGTTGTTTTG
ACCTGAATTGAGGTTAAGGAAGATTAAGCGTCAAACTTTGCGTGTGTGTGGGACCATTAG
CATGGGAGAACATGTAGAATGCAAAGAATATGTTTCAGACTTGACGTTAAGCTTTAGGTA
CTCTTCTCTTGCTCTCATGCCTATAAATATTATCCCTCATCAACTATAATTTTCACAGTCAA
AACACACACAATTGATTAAGAGAGCTTAATTTAGAGAGGAAAAGAAA**ATG**

Figure S9 the promoters and their motif of *NtGA3ox2* (A) and *NtGA2ox8* (B)
Yellow highlight indicates MYB binding motif, Red letter indicates light response element.

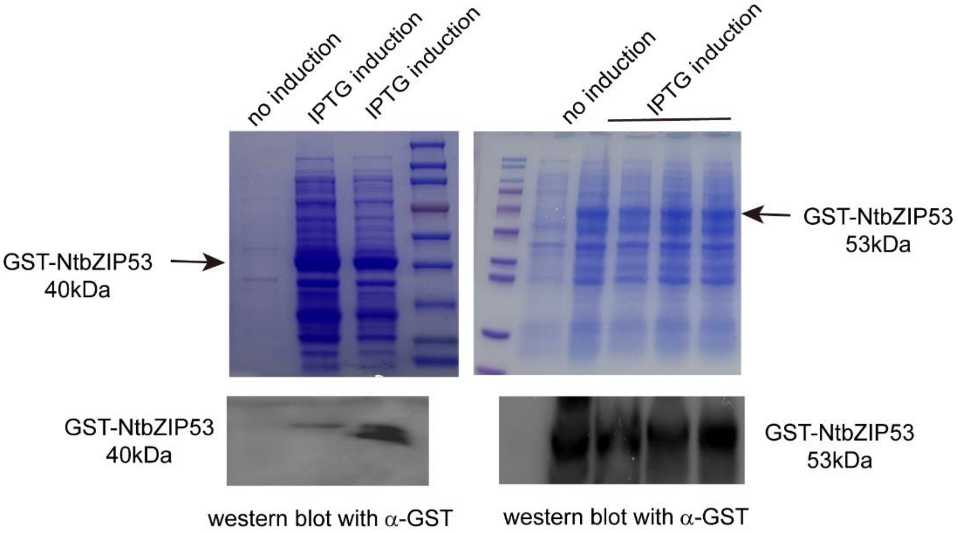

Figure S10 The recombinant proteins GST-NtbZIP53 and GST-NtMYB1 expressed in *E.coli*.

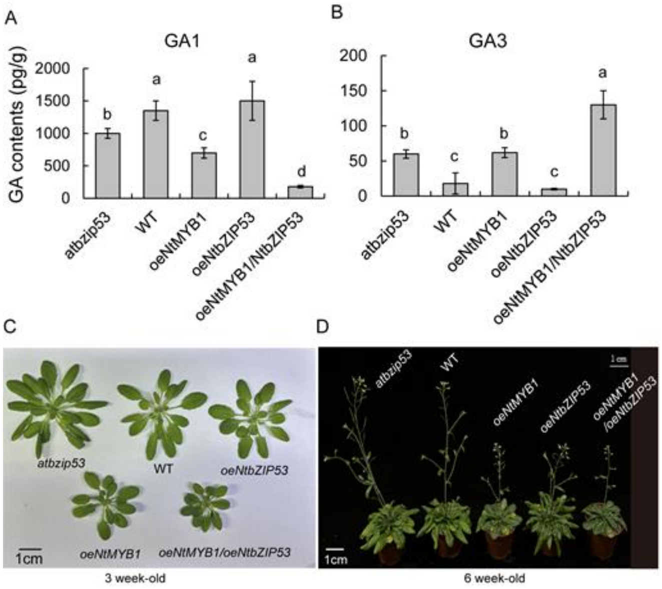

Figure S11 Overexpression of *NtMYB1* and *NtbZIP53* alters diverse GAs content and tiny-type in Arabidopsis. (A-B) The content of GA1 and GA3 in the *oeNtMYB1* or *oeNtbZIP53* or *oeNtMYB1/NtbZIP53* Arabidopsis leaves; (C-D) The tiny phenotype of the *oeNtMYB1* or *oeNtbZIP53* or *oeNtMYB1/NtbZIP53* Arabidopsis at 3-week-old (A) and at 5-week-old stage; Statistical analysis is done by Turkey's test, different letter indicates significant ($P<0.05$) (A-B)

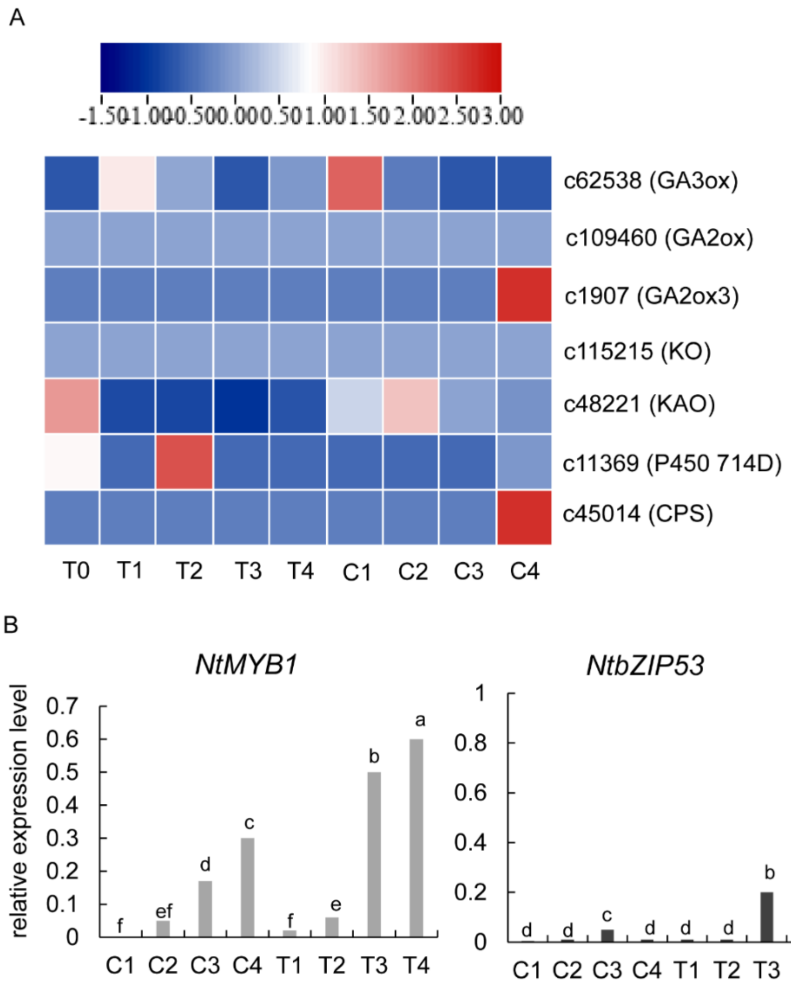

Figure S12 The expression profile of *NtGA3ox2, NtGA2ox8, NtMYB1* and *NtbZIP53* during narcissus development.

Table S1 The list of primer sequences in this study

**RT-qPCR primers**	
*NtMYB1*_F	AAGGCCCACACAAACAAAGG
*NtMYB1*_R	TCCAACAGCCTTCACCATGAG
*NtMYB1*_F2	ATGGGCAGAACTCCTTGTTG
*NtMYB1*_R2	CGTAAAATTACCCCGCTTGA
*AtGAPC2*_F	ACCACTGTCCACTCTATCACTGC
*AtGAPC2*_R	TGAGGGATGGCAACACTTTCCC
*NtActin*_F	GAGAGGGGTTACTCATTCAC
*NtActin*_R	CTCATAGCTCTTCTCCACTG
AtKS1_F	AAGGACGCAATGTGACACAC
AtKS1_R	TTGACTTGTCACTGGACCACTC
*AtGA20OX1*_F	ACATGAGCCGCTCAAAATCC
*AtGA20OX1*_R	TTCCCAAATGGCTGAAACCC
*AtGA20OX2*_F	AGAAGCTTGCACCAAACACG
*AtGA20OX2*_R	TCCATCAAACGGTGAGCATC
*AtGA20OX3*_F	AACGGAATGCATTGCAACGG
*AtGA20OX3*_R	TGGTGCGAGTGCTGATTAAG
*AtGA3OX1*_F	ACGCAAGCTTAAGTCTGCTC
*AtGA3OX1*_R	AACCTTCGGACCACATTTGC
*AtGA3OX2*_F	TGCAAAAGTTGGCAGCCAAG
*AtGA3OX2*_R	CGGCCCATTGTATGTCCTTTTC
*AtGA3OX4*_F	AGCTGGTGATGTGCAAATCG
*AtGA3OX4*_R	TAATTGCGTCCATGGCCTTG
*AtGA2OX2*_F	AAACGGATTGGACCAAACGG
*AtGA2OX2*_R	AAAACGGCGGAGGTTTTAGG
*AtGA2OX4*_F	ACATTGGACTCAACGGTGAC
*AtGA2OX4*_R	AACAATTCACTGCCGAGCTG
*AtGID1*_F	TTGTCGCGGGTTTGGATTTG
*AtGID1*_R	GGCAAGAGGTAAAACCCAACAG
AtGA3OX1_F	*ACGCAAGCTTAAGTCTGCTC*
AtGA3OX1_R	*AACCTTCGGACCACATTTGC*
AtGA3OX2_F	*TGCAAAAGTTGGCAGCCAAG*
AtGA3OX2_R	*CGGCCCATTGTATGTCCTTTTC*
AtGA20OX1- F	*CCACCGAGAGAGCTTTTGGA*
AtGA20OX1-R	*TGGGTTTGGTGAGCCAATCT*
AtGA200X3-F	*ATCAAGACCAAGTTGGCGGT*
AtGA200X3-R	*TAGAGCCATGAAGGTGTCGC*
**Subclone primers**	
ACT2pro: *NtMYB1*_F	gtgtgtgacctcgagactagtATGGGCAGAACTCCTTGTTGTG
ACT2pro: *NtMYB1*_R	ataccaggtggaggtcccccgggTCTAATTACATGTGGATTGGAAGACTTCA TTTGGC
35Spro: *NtMYB1*_F	caccATGGGCAGAACTCCTTGTTGTG

35Spro: *NtMYB1*_F	TCTAATTACATGTGGATTGGAAGACTTCATTTGGC
D37-NtbZIP53-FP	*TATCCAGATCCAGTGGGATCCATGTCTTCATTAACTCTCTGCCAATC*
D37-NtbZIP53-RP	*CGCACTAGTAAGCTTGGTACCGAACTGGAACATGTTTGCCATTAC*
AD-NtbZIP53-FP	*GCCATGGAGGCCAGTGAATTCATGTCTTCATTAACTCTCTGCCAATCC*
AD-NtbZIP53-RP	*CAGCTCGAGCTCGATGGATCCGAACTGGAACATGTTTGCCATTAC*
BD-NtbZIP53-FP	*ATGGCCATGGAGGCCGAATTCATGTCTTCATTAACTCTCTGCCAATCC*
BD-NtbZIP53-RP	*CCGCTGCAGGTCGACGGATCCGAACTGGAACATGTTTGCCATTAC*
YFPN/C-NtbZIP53-FP	*TCTGATCAAGAGACAGGATCCATGTCTTCATTAACTCTCTGCCAATC*
YFPN/C-NtbZIP53-RP	*TACAGTTCCACCTCCACTAGTGAACTGGAACATGTTTGCCATTAC*
YFPN/C-NtMYB1-FP	*TCTGATCAAGAGACAGGATCCATGGGCAGAACTCCTTGTTGTG*
YFPN/C-NtMYB1-RP	*TACAGTTCCACCTCCACTAGTTCTAATTACATGTGGATTGGAAGACTT*
pFmcherry-NtbZIP53-FP	*GTGTGTGACCTCGAGACTAGTATGTCTTCATTAACTCTCTGCC*
pFmcherry-NtbZIP53-RP	*GTCCTTGTAGTCCATACTAGTGAACTGGAACATGTTTGCCATTAC*
pTRV2-NtbZIP53-FP	*ATCGACGACAAGACCCTGCAGAAACAGGTTGCTCAACTTC*
pTRV2-NtbZIP53-RP	*TGAGGAGAAGAGCCCTGCAGGAACTGGAACATGTTTGCC*
pTRV2-NtMYB1-FP	*ATCGACGACAAGACCCTGCAGCTACTCAGCTGTGGAC*
pTRV2-NtMYB1-RP	*CTGAGGAGAAGAGCCCTGCAGTCTAATTACATGTGGATTGGAAG*
pGreen-NtGA30X2pro-FP	*CTATAGGGCGAATTGGGTACCAGGAGGCAGGCCATATGGTACT*
pGreen-NtGA30X2pro-RP	*CGTCTTCCATCTCGAGGGATCAAGGGTGGATCATGAGGGTGAT*
Genome-walking assay	
NtGA3OX2pro-1SP1	*ATCCATTCCCCGTCCTTGAGCAACTG*
NtGA3OX2pro-1SP2	*GCGTCGGTGTGAGCCCTTAAG*
NtGA3OX2pro-1SP3	*TCCCTTCTCTAGCCCTAGGTTCTC*
NtGA3OX2pro-2SP1	*GCGTCGGTGTGAGCCCTTAAG*
NtGA3OX2pro-2SP2	*TCCCTTCTCTAGCCCTAGGTTCTC*
NtGA3OX2pro-2SP3	*AAGGGTGGATCATGAGGGTGAT*
NtGA3OX2pro-3SP1	*AACAGCTCATGGGTGATCCCATGGTT*
NtGA3OX2pro-3SP2	*CCAGTTCTCGCAAGCATCTTGAAG*
NtGA3OX2pro-3SP3	*GCTTTGTGAGTTGCGCTATGTGCT*
pGreen-NtGA3OX2pro-FP	*CTATAGGGCGAATTGGGTACCAGGAGGCAGGCCATATGGTACT*
pGreen-NtGA3OX2pro-RP	*CGTCTTCCATCTCGAGGGATCAAGGGTGGATCATGAGGGTGAT*
NtGA2OX8pro-1SP1	*TCTCTCTGCCAGTTTCTCCAGC*
NtGA2OX8pro-1SP2	*TGGCGCAAGAAGAAAGTGCTCTCC*
NtGA2OX8pro-1SP3	*GTAGTGCTCCTTGGTCATTCTCTC*

NtGA2OX8pro-2SP1	*GGCATGAGAGCAAGAGAAGAGT*
NtGA2OX8pro-2SP2	*CACACACGCAAAGTTTGACGCT*
NtGA2OX8pro-2SP3	*GACCACCGACCACAGTATGATT*
NtGA2OX8pro-3SP1	*CGGTATACCTTCATAGAGGCAGATC*
NtGA2OX8pro-3SP2	*ACTCCAAAGTATCGGATTCGACCGAG*
NtGA2OX8pro-3SP3	*GGAACAGACATTGCATCGTTTGCATG*
pGreen-NtGA2OX8pro-FP	*CTATAGGGCGAATTGGGTACCCCGCCCTTTAAAAGTACAGT*
pGreen-NtGA2OX8pro-RP	*CGTCTTCCATCTCGAGGGATCCGACATTGCATCGTTTGCATG*
